# Supplementary material for: Intrinsically disordered protein PID‐2 modulates Z granules and is required for heritable piRNA‐induced silencing in the Caenorhabditis elegans embryo
Source: EMBO J. 2020 Nov 24;40(3):e105280. doi: 10.15252/embj.2020105280 (PMC7849312; doi:10.15252/embj.2020105280)
Supplement: Supplementary file 1 — Appendix [file EMBJ-40-e105280-s001.pdf]

Appendix to:

Intrinsically disordered protein PID-2 modulates Z granules and is required for heritable piRNA-induced silencing in the *C. elegans* embryo

Maria Placentino[1,2], António Miguel de Jesus Domingues[1], Jan Schreier[1,2], Sabrina Dietz[2,3], Svenja Hellmann[1], Bruno F. M. de Albuquerque[1,4], Falk Butter[2], René F. Ketting[1,5]

Institute of Molecular Biology (IMB), Ackermannweg 4, 55128, Mainz, Germany

[1] IMB, Biology of Non-coding RNA Group

[2] International PhD Programme on Gene Regulation, Epigenetics & Genome Stability, Mainz, Germany

[3] IMB, Quantitative Proteomics Group

[4] Graduate Program in Areas of Basic and Applied Biology, University of Porto, 4099-003 Porto, Portugal.

[5] Institute of Developmental Biology and Neurobiology, Johannes Gutenberg University, 55099, Mainz, Germany

correspondence: r.ketting@imb.de

*Contributed equally

Table of Contents **Page**

**1**	**Appendix Materials and Methods**	3-13
**2**	**Appendix Figures**	14-20
	Appendix Figure S1	14
	Appendix Figure S2	15
	Appendix Figure S3	16-17
	Appendix Figure S4	18
	Appendix Figure S5	19
	Appendix Figure S6	20

1 Appendix Materials and Methods

List of strains used in this study

Strain	Genotype
	wild type (Bristol N2)
RFK507	*pid-2(xf23) I*
RFK345	*pid-2(tm1614) I*
RFK231	*mjSi22[Pmex-5::mCherry::his-58::21UR-1_as::tbb-2(3'UTR)] I; mut-7(pk204) III; otIs45[Punc119::GFP] V*
RFK316	*mjSi22[Pmex-5::mCherry::his-58::21UR-1_as::tbb-2(3'UTR)] (RNAe) I; prg-1(n4357) I*
RFK851	*mjSi22[Pmex-5::mCherry::his-58::21UR-1_as::tbb-2(3'UTR)] I; prg-1(n4357) I*
RFK677	*pid-2(xf23) I; mjSi22[Pmex-5::mCherry::his-58::21UR-1_as::tbb-2(3'UTR)] I*
RFK585	*pid-2(xf23) I; mjSi22[Pmex-5::mCherry::his-58::21UR-1_as::tbb-2(3'UTR)] (RNAe) I*
RFK528	*pid-2(tm1614) I; mjSi22[Pmex-5::mCherry::his-58::21UR-1_as::tbb-2(3'UTR)] I*
RFK530	*pid-2(tm1614); mjSi22[Pmex-5::mCherry::his-58::21UR-1_as::tbb-2(3'UTR)] (RNAe) I*
RFK586	*pid-2(tm1614); mjSi22[Pmex-5::mCherry::his-58::21UR-1_as::tbb-2(3'UTR)] (RNAe) I*
SX2078	*mjSi22[Pmex-5::mCherry::his-58::21UR-1_as::tbb-2(3'UTR)] I*
RFK416	*mjSi22[Pmex-5::mCherry::his-58::21UR-1_as::tbb-2(3'UTR)] I; hrde-1(tm1200) III*
RFK587	*pid-2(tm1614) I; prg-1(n4357) I*
RFK709	*pid-2(xf23) I*
NL3643	*unc-22(st136::Tc1) IV*
RFK611	*wago-1(tm1414) I; wago-2(tm2686) I; ppw-2(tm1120) I; unc-22(st136::Tc1) IV*
RFK610	*pid-2(xf23) I; unc-22(st136::Tc1) IV*
RFK612	*pid-2(xf23) I; prg-1(n4357) I; unc22(st136::Tc1) IV*
RFK614	*pid-2(xf23) I; hrde-1(tm1200) III; unc22(st136::Tc1) IV*
RFK616	*pid-2(tm1614) I; unc22(st136::Tc1) IV*
RFK617	*pid-2(tm1614) I; prg-1(n4357) I; unc22(st136::Tc1) IV*
RFK619	*pid-2(tm1614) I; hrde-1(tm1200) III; unc22(st136::Tc1) IV*
RFK247	*prg-1(n4357) I; unc-22(st136::Tc1) IV*
RFK253	*hrde-1(tm1200) III; unc-22(st136::Tc1) IV*
SX523	*prg-1(n4357) I*
RFK315	*mjSi22[Pmex-5::mCherry::his-58::21UR-1_as::tbb-2(3'UTR)] I; pid-1(xf35) II; otIs45[unc-119::GFP] V*
RFK804	*pid-2(xf23) I; mjSi22[Pmex-5::mCherry::his-58::21UR-1_as::tbb-2(3'UTR)] I; pid-1(xf35) II; otIs45[unc-119::GFP] V*
RFK182	*pid-1(xf35) II*
RFK774	*pid-2(xf23) I; pid-1(xf35) II*
RFK525	*xfSi83[Pgl-3(5'UTR)::3xFLAG::pid-2::tbb-2(3'UTR); cb-unc119(+)] II*
RFK527	*xfSi98[Pgl-3(5'UTR)::3xFLAG::pid-2::tbb-2(3'UTR); cb-unc119(+)] V*
RFK504	*pid-2(xf23) I; xfSi83[Pgl-3(5'UTR)::3xFLAG::pid-2::tbb-2(3'UTR); cb-unc119(+)] II; mjSi144[Pmex-5::egfp::his-58::21UR-1_as::tbb-2(3'UTR)] II*
RFK505	*pid-2(xf23) I; xfSi98[Pgl-3(5'UTR)::3xFLAG::pid-2::tbb-2(3'UTR); cb-unc119(+)] V; mjSi144[Pmex-5::egfp::his-58::21UR-1_as::tbb-2(3'UTR)] II*
RFK654	*xfSi144[pid-2(5'UTR)::eGFP::pid-2::pid-2(3'UTR); cb-unc119(+)] II*
RFK655	*xfSi145[pid-2(5'UTR)::pid-2::eGFP::pid-2(3'UTR); cb-unc119(+)] II*

RFK656	*xfSi146[pid-2(5'UTR)::3xFLAG::pid-2::pid-2(3'UTR); cb-unc119(+)] II*
RFK693	*pid-2(xf23) I; xfSi144[pid-2(5'UTR)::eGFP::pid-2::pid-2(3'UTR); cb-unc119(+)] II*
RFK694	*pid-2(xf23) I; xfSi145[pid-2(5'UTR)::pid-2::eGFP::pid-2(3'UTR); cb-unc119(+)] II*
RFK695	*pid-2(xf23) I; xfSi146[pid-2(5'UTR)::3xFLAG::pid-2::pid-2(3'UTR); cb-unc119(+)] II*
RFK853	*mjSi22[Pmex-5::mCherry::his-58::21UR-1_as::tbb-2(3'UTR)] I pid-2(xf23) I; xfIs144[pid-2(5'UTR)::eGFP::pid-2::pid-2(3'UTR); cb-unc119(+)] II*
RFK854	*mjSi22[Pmex-5::mCherry::his-58::21UR-1_as::tbb-2(3'UTR)] I pid-2(xf23) I; xfSi145[pid-2(5'UTR)::pid-2::eGFP::pid-2(3'UTR); cb-unc119(+)] II*
RFK855	*mjSi22[Pmex-5::mCherry::his-58::21UR-1_as::tbb-2(3'UTR)] I pid-2(xf23) I; xfIs146[pid-2(5'UTR)::3xFLAG::pid-2::pid-2(3'UTR); cb-unc119(+)] II*
RFK184	*mjSi22[Pmex-5::mCherry::his-58::21UR-1_as::tbb-2(3'UTR)] I; pid-1(xf35) II*
RFK422	*mjSi22[Pmex-5::mCherry::his-58::21UR-1_as::tbb-2(3'UTR)] I (RNAe); pid-1(xf35) II*
RFK764	*mjSi22[Pmex-5::mCherry::his-58::21UR-1_as::tbb-2(3'UTR)] I; pid-1(xf35) II*
RFK765	*mjSi22[Pmex-5::mCherry::his-58::21UR-1_as::tbb-2(3'UTR)] I; pid-1(xf35) II*
RFK766	*mjSi22[Pmex-5::mCherry::his-58::21UR-1_as::tbb-2(3'UTR)] I; pid-1(xf35) II*
RFK767	*mjSi22[Pmex-5::mCherry::his-58::21UR-1_as::tbb-2(3'UTR)] I (RNAe); pid-1(xf35) II*
RFK768	*mjSi22[Pmex-5::mCherry::his-58::21UR-1_as::tbb-2(3'UTR)] I (RNAe); pid-1(xf35) II*
RFK769	*mjSi22[Pmex-5::mCherry::his-58::21UR-1_as::tbb-2(3'UTR)] I (RNAe); pid-1(xf35) II*
RFK771	*mjSi22[Pmex-5::mCherry::his-58::21UR-1_as::tbb-2(3'UTR)] I; pid-1(xf35) II; hrde-1(tm1200) III*
RFK857	*pid-4(xf184) I*
RFK858	*pid-4(xf185) I*
RFK859	*pid-5(xf181) V*
RFK860	*pid-5(xf182) V*
RFK847	*pid-4(xfSi186[pid-4::3xMyc]) I*
RFK879	*pid-5(xfSi192[pid-5::2xHA]) V*
RFK932	*pid-4(xfSi204[pid-4::d10]) I*
RFK988	*pid-4(xfSi206[pid-4::mTagRFP-T]) I*
RFK972	*pid-5(xfSi221[pid-5::d10]) V*
RFK1025	*pid-5(xfSi226[pid-5::mTagRFP-T]) V*
RFK982	*pid-4(xf184) I; mjSi22[Pmex-5::mCherry::his-58::21UR-1_as::tbb-2(3'UTR)] I*
RFK983	*pid-4(xf184) I; mjSi22[Pmex-5::mCherry::his-58::21UR-1_as::tbb-2(3'UTR)] (RNAe) I*
RFK984	*mjSi22[Pmex-5::mCherry::his-58::21UR-1_as::tbb-2(3'UTR)] I; pid-5(xf181) V*
RFK985	*mjSi22[Pmex-5::mCherry::his-58::21UR-1_as::tbb-2(3'UTR)] I; pid-5(xf181) (RNAe) V*
RFK986	*pid-4(xf184) I; pid-5(xf181) V*
RFK987	*pid-4(xf184) I; mjSi22[Pmex-5::mCherry::his-58::21UR-1_as::tbb-2(3'UTR)] I; pid-5(xf181) V*
RFK979	*hrde-1(tm1200) III*
RFK1085	*xfSi145[pid-2(5'UTR)::pid-2::eGFP::pid-2(3'UTR); cb-unc119(+)] II; pgl-1(xfSi233[pgl-1::mTagRFP-T]) IV*
RFK1194	*deps-1(ax2063[deps-1::GFP]) I; pid-4(xfSi206[pid-4::mTagRFP-T]) I*
RFK1195	*deps-1(ax2063[deps-1::GFP]) I; pid-5(xfSi226[pid-5::mTagRFP-T]) V*
RFK1119	*pid-4(xfSi206[pid-4::mTagRFP-T]) I; znfx-1(gg544[3xFLAG::GFP::znfx-1]) II*
RFK1118	*znfx-1(gg544[3xFLAG::GFP::znfx-1]) II; pid-5(xfSi226[pid-5::mTagRFP-T]) V*

RFK1186	*pid-4(xfSi206[pid-4::mTagRFP-T]) I; xfSi145[pid-2(5'UTR)::pid-2::eGFP::pid-2(3'UTR); cb-unc119(+)] II*
RFK1078	*xfSi145[pid-2(5'UTR)::pid-2::eGFP::pid-2(3'UTR); cb-unc119(+)] II; pid-5(xfSi226[pid-5::mTagRFP-T]) V*
RFK1184	*znfx-1(gg544[3xFLAG::GFP::znfx-1]) II; pgl-1(xfSi233[pgl-1::mTagRFP-T]) IV*
RFK1185	*pid-2(xf23) I; znfx-1(gg544[3xFLAG::GFP::znfx-1]) II; pgl-1(xfSi233[pgl-1::mTagRFP-T]) IV*
RFK1216	*pid-4(xf184) I; znfx-1(gg544[3xFLAG::GFP::znfx-1]) II; pgl-1(xfSi233[pgl-1::mTagRFP-T]) IV; pid-5(xf181) V*
RFK1217	*pid-4(xf184) I; znfx-1(gg544[3xFLAG::GFP::znfx-1]) II; pgl-1(xfSi233[pgl-1::mTagRFP-T]) IV*
RFK1218	*znfx-1(gg544[3xFLAG::GFP::znfx-1]) II; pgl-1(xfSi233[pgl-1::mTagRFP-T]) IV; pid-5(xf181) V*
RFK1277	*pid-4(xfSi206[pid-4::mTagRFP-T]) I; simr-1(cmp1022[simr-1::GFP3xFLAG]) I*
RFK1288	*simr-1(cmp1022[simr-1::GFP3xFLAG) I; pid-5(xfSi226[pid-5::mTagRFP-T]) V*
RFK1050	*pid-4 (xf184) I; pid-5(xf181) I; mjSi22 [Pmex-5::mCherry::his-58::21UR-1_as::tbb-2(3'UTR)] [RNAe] I*

Small RNA sequencing

Small RNA enrichment

In order to enrich for small RNAs, we used the *mir*Vana™ kit (Art. No. AM1561, Invitrogen™). 400 µl of *mir*Vana™ Lysis/Binding buffer and 48 µl of *mir*Vana™ Homogenate Additive have been added to the total RNA (80 µl). The mix has been incubated at RT for 5 minutes to denature RNA, then 1/3 of volume of 100% ethanol has been added and after mixing, samples have been spun down at 2500 x g for 4 minutes at RT to pellet large RNAs (>200 nt). The supernatant has been transferred to a new Eppendorf tube and RNA has been precipitated at -80 °C for 1 h with isopropanol (1:1). Samples have been centrifuged at maximum speed for at least 10 minutes at 4 °C to pellet small RNAs. The pellet has then been washed twice with 75% ethanol and spun down at maximum speed for 5 minutes at 4 °C. Pellet has been dried and resuspended in 16 µl of nuclease-free water. RNA quality has been checked at Nanodrop and on agarose gel and further processed for library preparation and deep sequencing.

Small RNA classification and quantification

Gene annotation was retrieved from Ensembl (release-38) and merged with transposon coordinates, retrieved from Wormbase (PRJNA13758.WS264), creating a custom annotation used for the analysis. Non-strutural mapped reads were categorized in small RNA classes as follows: 21U RNAs are 21 nt long sequences with a 5'U mapping sense to annotated 21U RNA loci; 22G RNAs are 20-23 nt long with a 5'G and map antisense to protein-coding/pseudogenes/lincRNA/transposons; 26G RNAs, are those which are 26 nt long with a 5'G, and map antisense to annotated protein-coding/pseudogenes/lincRNA; and miRNAs are 20-24 nt long mapping sense to annotated miRNA loci. Read filtering was done with a python script (https://github.com/adomingues/filterReads/blob/master/filterReads/filterSmallRNAclasses.py) based on pysam v0.8.1 / htslib (Li et al., 2009), in combination with Bedtools intersect. For miRNA a stricter intersection was required (intersectBed -f 1.0). Reads belonging to each class were then counted for each library (total levels). For the analysis of small RNA classes, previously published gene lists were used: ALG-3/-4 (Almeida et al., 2019a); ERGO-1 (Almeida et al., 2019a); CSR-1 (Conine et al., 2013); NRDE-3 (Zhou et al., 2014); mutators (Phillips et al., 2014); and WAGO-1 (Gu et al., 2009). The

genomic locations of 22G were then intersected with that of the genes, counted for each library and normalized to non-structural reads.

22G RNAs coverage on 21U sensor

For targeting of the 21U sensor by 22G RNAs, we considered only sequences that were 22 nt long and mapping unambiguously to the 21U sensor sequence. Coverage was calculated with Bedtools v2.25.0 (genomeCoverageBed -ibam -d -strand "-") (Quinlan & Hall, 2010). Visualization was created with the R/Bioconductor package ggbio (Yin et al., 2012).

Secondary and tertiary 22G RNAs on 21U sensor

Tertiary populations were defined as the 22G RNAs mapping antisense to the mCherry coding sequence, within the 21U sensor (Sapetschnig et al., 2015). To define the secondary 22G (Sapetschnig et al., 2015), those surrounding the *21ur-1* recognition site, the spread from this site was visually estimated on a genome browser (IGV) to be +/- 200 bp. The reads mapping to these positions were counted and normalized to non-structural reads.

Coverage of local 22G RNAs on endogenous 21U RNA target sites

Following the analysis of (Lee et al., 2012), 21U RNA targets were identified by mapping the annotated 21U RNA sequences (WBCel235) to the genome with bowtie v1.2.1.1 (-n 1 -l 8 -e 300 -k 1000 –best), allowing for one mismatch in the 8 nucleotide seed and reporting up to 1000 valid alignments. The alignments were then filtered allowing for one T to G change in the seed, and two additional mismatches and one T to G change in the remaining alignment (see also Bagijn et al., 2012). These putative 21U RNA target coordinates were then intersected with the genomic location of WAGO-1 associated genes, to define a confident set of 21U RNA binding sites. Finally the 5' 21U RNA mapping site was shifted by 10 nt (bedtools shift -s 10), extended in both directions by 50 bp (slopBed -l 50 -r 50 -s), and finally the 22G RNA coverage was calculated for these regions (coverageBed -d -s), and normalized by the number of non-structural reads.

Differential small RNA targeting

22Gs mapping to annotated features in the custom GTF were counted with htseq-count v0.9.0 (htseq-count -s reverse -f bam -m intersection-nonempty). Differential expression comparisons were performed with DESeq2 v.1.18.1 (Love et al., 2014). For the selection of genes differentially targeted (mRNA), a cut-off of at least a two-fold-change difference between conditions and an adjusted p-value less than 0.05 was applied.

Replication histone gene IDs were retrieved from Wormbase, divided into groups (H2A, H2B, H3, H4), and the 22G read counts each group extracted from the counts tables generated with htseq-count. 22Gs read counts where then summed for each group and normalized to non-structural reads. Statiscal comparison was performed with the Wilcoxon signed-rank test.

Overlap with siRNA pathways

To determine if genes whose expression is affected in the *pid-2* mutants are shared with other siRNA pathways, we collected list of genes previously identified as being targeted by the following pathways: CSR-1 (Conine et al., 2013); NRDE-3 (Zhou et al., 2014); mutators (Phillips et al., 2014); WAGO-1 (Gu et al., 2009); ERGO-1 and ALG-3/-4 (Almeida et al., 2019a). ALG-3/-4 targets do not include those shared with ERGO-1 targets. See also Almeida at al., 2019a. Gene overlap significance was determined with the R/Bioconductor package GeneOverlap, and p-values calculated with Fisher's exact test.

22G siRNA coverage

For the metagene analysis with 22G coverage over genes, we broadly followed the analysis described in (Ishidate et al., 2018). Briefly, 22G coverage, normalized to mapped non-structural reads, was plotted along the gene bodies with DeepTools computeMatrix scale-regions —metagene —transcriptID gene —transcript_id_designator gene_id —missingDataAsZero -b 0 -a 0 —regionBodyLength 2000 —binSize 20 —averageTypeBins mean. The resulting coverage matrix was used to produce the final plots in R. Coverage in each bin is normalized to the total 22G coverage of the gene. Genes with total coverage less than 5 were removed. For statistical analysis of 22Gs coverage over the gene body, genes were diving into 10 bins, and coverage compared between wild-type and mutant with a two-sided Student's t-Test and p-values adjusted the bonferroni method.

<u>21U mCherry sensor sequence</u>

2246-2733: mex-5; 2734-3640: mCherry; 3641-4012: his-58; 4040-4061: 21U; 4062-4393: tbb-2_UTR

ctttgagccaatttatccaagtccttgtaaaaagtattcgaaaattgttaacggataaaatgtttattataatatcaaaaacaatttgtcagttgacc
actttttgatatatttgacagaaaccgggatgaattggctcaaagttagggcgcccttctattacagggtttctgataacaaaccggttattaactc
ccaacaagggatgatttcaattcatcatgctcaaattgacccaaattaagttacatgacaaattcatcgcccttttcaactcttcttggctcatcatc
tgttattctgttcctattctgtctgcaccctataccccttttgcatactctctcgtcattcctttcttggatagtgtcttcttctccccagctctgctacttct
atgacttgcgcccgctgcttttccgctcgttcctctcctctctgacgtcttcttctgcttcttttctccttcctttttcccttctcaatcttcctctttttccca
tttctgtcaatcattcgaagaagaagaagaagaagaccctcattcatttcatttttttttctgtcggtgtgtgctgctcggttaagagtgagctctcta
ttccacgtcttcttctttttcttcttcgattcgaatcaatcactccaccaaaacgcattcgtttttgggattcaccccgcggttcgcaaTaggtttctttt
tcaaattattagcgttataaatagaaaaatggtggagtttcaataaaaatgataatttacaaagtgtattttgattacatgtactcaaaaaggttga
aatttttcataccagttttccggaaatccatctgatatcattatcgtatttttctttttaaaaatgttttcaaaaaaaaacaaaatatagctggttattt
ggcaccctctaattaccattttcctgtcacaccacactcttttccttcttccacttctttccgttttcagccgcttccaaccaaaccgatatgaaagccg
agcaacaacaatcgattccacccggctcggcgaccttcccgtcgcaggtgagactcagaaaactagagaaaccgctcaactaactcttgatatcc
gatttcattcttttctcttttctttttgttgaacttttccatatttccagatgccacggccaccaccaagcaccgaacaaggaatcacaacggaatcgg
agcttgcgaagaaagctcaaatcactccgaacgacgttttagcacttccgggaatcactcaaggtatcttgctccctttggatttcagtttaaaaca
taaatttaggattcttatgctccccatctgcgaacatctataacatcgagttcaccaagttccaaatccgtgatctggacactgagcaagtgcttttc
gagatcgccaaaccggagaacgatcaggagaatgatgagtcgccacaggagtcggcaagatacgtgcgttatagatttgctccaaactttttga
aactcaaaacggtcggagcaactgtggaattcaaagtaggagacatcccaatccatcatttccgaatgatcgaacgtcacttcttcaaagatcgc
cttctgaagtgttttgactttgaattcggattctgtattccgaattcacgaaacaactgtgaacatatctatgagttccctcaactctctcaacaactc
agtgagtcattattctaaaaagtacaattcaaaagactaatctcttttttcagtggacgacatgatcaacaatccaaacgagactcgttctgacagc
ttctatttcgtcgataacaaactcgtcatgcacaacaaagccgactactcatatgatgcataaatatttaatacaaaaatgttctggataattattct
gtcgaatagaaaaaaaactccaaatgtgattaaattccaataattcctgtctagtttgttccttccccttccccttctcatgttcaatgcattcctaag
cttttcagttccccccttgtttctatatttttttcgctgtcctgtcacactcgctaaaaacactaatcacacggaaatctgttttcaataaaaactccaac
tttaactcattttcaatttcaactgaaagatttttttcattagagaatgtctagaactagGCCCGGGCTACGTAATACGACTCACTTAAG
GCCTTGACTAGAGGGTACCAGAGCTCACCTAGGcaggaacagctatgaccatgattacgccaagctatcaactttGTATAGAA
AAGTTGAAATATCAGTTTTTAAAAAATTAAACCATAAAACAAATAATATAACCCAATTTTTACATCAAACCACAA
GAAAAAAATACATTTGGGCCCACGGATAAAGAAATTAAAAAAATACATTTTTTAAAGGCGCACCGAATTAAAA
TTCATTTGGGTCTTACCGCGTATACCGTACTCCGTTTGTTTGATCATTTTTGTCAGCGCTGGCGGTTGTTTTTTCA
TTTCATTTCTGCTTCAAAGACGTTTTCTCGAATAATTTTTCGTTTATTCTCTTTTTTAAAATTAATTTCTAGCCGTA
AATGTTATAAATTCACCCATTTAACGCAAATTTCATGGTAATCTCATGGAAAAATGCAGTTTCTTTGTTAAAGAA
AGCTTAAATAGCAAAAATTCCCCGACTTTCCCCAAAATCCTGCTCGATTTTCCGTTTTCTCATTGTATTCTCTCTT
AATTAATTTTATCGATAATCAATTGAATGTTTCAGACAGAGAATGGTCTCAAAGGGTGAAGAAGATAACATGG
CAATTATTAAAGAGTTTATGCGTTTCAAGGTGCATATGGAGGGATCTGTCAATGGGCATGAGTTTGAAATTGA
AGGTGAAGGAGAAGGCCGACCATATGAGGGAACACAAACCGCAAAACTAAAGgtaagtttaaacatatatatactaac
taaccctgattatttaaattttcagGTAACTAAAGGCGGACCATTACCATTCGCCTGGGACATCCTCTCTCCACAGTTCAT
GTATGGAAGTAAAGCTTATGTTAAACATCCGGCAGATATACCAGATTATTTGAAACTTTCATTCCCGGAGGGTT
TTAAGTGGGAACGCGTAATGAATTTTGAAGACGGAGGAGTTGTTACAGTGACGCAAGACTCAAGgtaagtttaaa
cagttcggtactaactaaccatacatatttaaattttcagCCTCCAAGATGGAGAATTTATTTATAAAGTCAAACTTCGAGGAA

CGAATTTCCCCTCGGATGGACCTGTTATGCAGAAGAAGACTATGGGATGGGAAGCTTCAAGTGAAAGAATGT
ACCCTGAAGACGGTGCTCTTAAGGGAGAGATTAAACAACGTCTTAAATTGAAAGATGGAGGACATTACGATGC
TGAGgtaagtttaaacatgattttactaactaactaatctgatttaaattttcagGTGAAGACAACTTACAAAGCCAAAAAACCAG
TTCAGCTGCCAGGAGCGTACAATGTTAATATTAAACTGGATATCACCTCCCACAACGAGGATTACACTATCGTT
GAGCAATATGAAAGAGCTGAAGGGCGGCACTCGACAGGTGGCATGGATGAATTGTATAAGGGAGGTGGAGG
TGGAGCTTCAAGTTTGTACAAAAAAGCAGGCTCGATGCCACCAAAGCCATCTGCCAAGGGAGCCAAGAAGGC
CGCCAAGACCGTCGTTGCCAAGCCAAAGGACGGAAAGAAGAGACGTCATGCCCGCAAGGAATCGTACTCCGT
CTACATCTACCGTGTTCTCAAGCAAGTTCACCCAGACACCGGAGTCTCCTCCAAGGCCATGTCTATCATGAACTC
CTTCGTCAACGATGTATTCGAACGCATCGCTTCGGAAGCTTCCCGTCTTGCTCATTACAACAAACGCTCAACGAT
CTCATCCCGCGAAATTCAAACCGCTGTCCGTTTGATTCTCCCAGGAGAACTTGCCAAGCACGCCGTGTCTGAGG
GAACCAAGGCCGTCACCAAGTACACTTCCAGCAAGTAAACCCAGCTTTCTTGTACAAAGTGGtcaagcacggttaac
gtacgtaccaGATAAATGCAAAATCCTTTCAAGCATTCCCTTCTTCTCTATCACTCTTCTTTCTTTTTGTCAAAAAATT
CTCTCGCTAATTTATTTGCTTTTTTAATGTTATTATTTTATGACTTTTTATAGTCACTGAAAAGTTTGCATCTGAGT
GAAGTGAATGCTATCAAAATGTGATTCTGTCTGATGTACTTTCACAATCTCTCTTCAATTCCATTTTGAAGTGCTT
TAAACCCGAAAGGTTGAGAAAAATGCGAGCGCTCAAATATTTGTATTGTGTTCGTTGAGTGACCCAACAAAAA
GAGGAAACTTTATTGTGCCGCCAAGAAAAAAGTCTCACAACTTTATTATACAAAGTTGataattcactggccgtcgtttt
acaCTCGAGACGTACGGTGCGCGCGATGCATTCGAAGATCTGCCCACTAGTGAGTCGTATTA

Production of PID-2 protein for antibody generation

Cloning

In order to produce a polyclonal antibody against PID-2, we cloned the full-length coding sequence (CDS) of *pid-2* (UniProtID Q9N3P1) into a vector for recombinant protein overexpression in *E. coli*.

The cDNA, obtained from wild type worms with the ProtoScript® First Strand cDNA Synthesis Kit (Art. No. E6300S, New England Biolabs), has been used as template for the amplification of the CDS of *pid-2* using Q5® Hot Start High-Fidelity DNA Polymerase (Art. No. M0493, New England Biolabs). PCR primers were designed to contain restriction sites (forward: NcoI ggccatgggcatgacagttattatagcgtcacact; reverse (without stop codon): XhoI ggctcgagaaatggg cactcgctgaat) for subsequent cloning into the pET-28a(+) expression vector (Art. No. 69864-3, Merck), which confers bacterial resistance to Kanamycin and adds a 6x histidine tag for affinity purification, either at the N- or at the C-terminus of the protein of interest. The PCR product was purified from agarose gel (QIAquick Gel Extraction Kit, Art. No. 28706, QIAgen). The vector and the PCR product (insert) have been digested with the restriction enzymes NcoI (Art. No. R3193, New England Biolabs) and XhoI (Art. No. R0146, New England Biolabs) for 2 hours at 37 °C. The insert has been additionally dephosphorylated using Antarctic Phosphatase (Art. No. M0289, New England Biolabs) for 15 minutes at 37 °C to avoid self-ligation. Ligation was then performed for 30 minutes at room temperature using a molar ratio 3:1 = vector:insert using T4 DNA Ligase (Art. No. M0202, New England Biolabs). The ligation reaction was then transformed in Subcloning Efficiency™ DH5α™ Competent Cells (Art. No. 18265017, Invitrogen™) and plated for selection on LB agar plates with Kanamycin (30 µg/ml). The plasmid was isolated from the bacterial culture (PureLink™ HiPure Plasmid Miniprep Kit, Art. No. K210011, Invitrogen™), checked by enzymatic digestion and sequencing.

Protein expression and purification

For protein expression, the pET-28a(+)-PID-2 construct has been transformed into Rosetta™ (DE3) Competent Cells (Art. No. 70954-3, Merck) and positive clones have been selected on LB agar plates with Kanamycin (30 µg/ml). A single colony has been inoculated into 20 ml of LB media supplied with 100 µg/ml Kanamycin and 35 µg/ml Chloramphenicol as pre-culture and grown overnight at 37 °C. The pre-culture has then been inoculated in 1 l LB media supplied with 100 µg/ml Kanamycin and 35 µg/ml Chloramphenicol and grown for 3 hours at 37 °C, until exponential phase (OD600 = 0,6). Protein

expression has been induced by adding 0,5 mM IPTG (Art. No. V3953, Promega) and incubated overnight at 18 °C. The bacterial culture has been harvested by spinning down at 4000 x g for 15 minutes at 4 °C. Cells pellet has been collected and washed with HEPES buffer to remove excess of growth media. The bacteria were spun down again at 4500 x g for 20 minutes at 4 °C and the pellet was frozen at -20 °C. In order to purify PID-2 protein tagged with 6xHis-tag from inclusion bodies, bacterial pellet was thawn on ice and resuspended in 30 ml of lysis buffer (500 mM NaCl; 100 mM Tris HCl pH=8.5). The pellet has been homogenized, sonicated 3 x 3 minutes (Branson Sonifier 450; output 4-5; duty cycle 2-3) and the cell lysate has been centrifuged at 19000 x g for 25 minutes at 4 °C. The pellet containing PID-2 has then been resuspended in 30 ml of denaturing buffer (500 mM NaCl; 100 mM Tris HCl pH=8.5; 8 M urea) to solubilize the inclusion bodies and centrifuged again at 19000 x g for 25 minutes at 4 °C to remove cells debris. The supernatant has then been loaded on a batch column containing 1 ml of Ni-NTA Agarose slurry (Art. No. 30210, QIAgen), previously equilibrated with the same buffer (500 mM NaCl; 100 mM Tris HCl pH=8.5; 8 M urea). After binding of the protein, the Ni-NTA Agarose beads have been washed with 10 ml of buffer and then eluted with 10 ml of elution buffer (500 mM NaCl; 100 mM TrisHCl pH=8.5; 4 M Urea; 250 mM imidazole). Eluate was stored at -80 °C.

<u>Antibody production</u>

After checking the purity of the protein by SDS-PAGE, the eluted protein PID-2::6xHis has been concentrated to a final concentration of 1,8 mg/ml and sent to Eurogentec for antibody production (two rabbits; 28-day Speedy protocol). We then received the serum from two rabbits (823 and 824) and used 823 for all the experiments (1:100 for immunoprecipitation).

Transgenic lines generation using the miniMos system

We generated *pid-2* transgenic lines using the miniMos system, as previously described (Frøkjær-Jensen et al., 2014). The injection mix contains plasmids encoding for the co-injection markers (10 ng/µl pGH8; 2,5 ng/µl pCFJ90; 5 ng/µl pCFJ104), for the transposase (50 ng/µl pCFJ601) and for the desired transgene embedded in a modified Mos element (10 ng/µl pRK1012). We injected the mix in the strain HT1593, which carries an *unc-119(ed3) III* mutation, and kept the worms at 25 °C until starvation. We first screened for mCherry expressing worms, indicative of a successful injection, and later on, for wild-type moving worms that have no extrachromosomal array (no mCherry expression), indicating that the *unc-119(ed3) III* mutation has been rescued by the wild-type allele encoded by the template plasmid pRK1012. We have isolated seven independent insertions of the desired transgene. Worms were then lysed and genotyped to confirm the insertion of the transgene. Afterwards, we mapped the insertion of the transgenes, using an inverse PCR approach, as previously described (Frøkjær-Jensen et al., 2014). We managed to map only two insertions, *xfSi83* and *xfSi98*. The insertion of the transgene *xfSi83* was mapped to chromosome II, within the last intron of *mpz-1*, whereas the transgene *xfSi98* was mapped to chromosome V, within the fourth intron of the Y32B12B.4 gene.

Transgenic lines generation using the MosSCI system

In order to avoid potential variability effects on the expression of the *pid-2* transgenes generated with the miniMos system, due to their insertion locus, we generated *pid-2* transgenic lines, using the MosSCI system and targeted the locus *ttTi5605* on LGII, as previously described (Frøkjær-Jensen et al., 2008). The injection mix contains plasmids encoding for the co-injection markers (10 ng/µl pGH8; 2,5 ng/µl pCFJ90; 5 ng/µl pCFJ104), for the transposase (50 ng/µl pCFJ601) and for the desired transgene (50 ng/µl pRK1036, pRK1037 or pRK1038). We injected the mix in the strain EG6699, which carries a Mos insertion on the locus *ttTi5605* on LGII, and kept the worms at 25 °C until starvation. We first screened for mCherry expressing worms, indicative of a successful injection, and later on, for wild-type moving worms that have no extrachromosomal array (no mCherry expression). From the templates pRK1036,

pRK1037 and pRK1038, we have isolated *xfSi144* [eGFP::PID-2], *xfSi145* [PID-2::eGFP] and *xfSi146* [3xFLAG::PID-2], respectively. Worms were then lysed and genotyped to confirm the insertion of the transgene.

Tc1 reversion data

Experiment	Genotype	T	R	N	f
1	*wt*	20	0	10,000	0
1	*wago-1/2/3*	15	4	1,000	0.00031
1	*prg-1*	20	0	10,000	0
1	*hrde-1*	20	5	10,000	0.000029
1	*pid-2*	20	3	10,000	0.000015
1	*pid-2; prg-1*	20	0	10,000	0
1	*pid-2; hrde-1*	20	19	4,000	0.00075
2	*wt*	20	0	10,000	0
2	*wago-1/2/3*	20	7	1,000	0.00043
2	*prg-1*	20	0	10,000	0
2	*hrde-1*	20	7	10,000	0.000043
2	*pid-2*	35	8	10,000	0.000026
2	*pid-2; prg-1*	35	0	10,000	0
2	*pid-2; hrde-1*	22	16	2,000	0.00065

Generation of mutant and endogenously tagged lines using CRISPR/Cas9 technology

Generation of mutant lines

Wild type worms have been injected with an injection mix containing 50 ng/µl pJW1259 (encoding for *Peft-3::cas9::tbb-2 3'UTR*, a gift from Jordan Ward (Ward, 2015)), co-injection markers (10 ng/µl pGH8; 5 ng/µl pCFJ104; 2,5 ng/µl pCFJ90) and 30 ng/µl of each of the plasmids encoding for the sgRNAs, specifically pRK1054, pRK1056, pRK1057, pRK1059 to target the *Y45G5AM.2* locus and pRK1047, pRK1050, pRK1052, pRK1053 to target the *W03G9.2* locus. After injections, worms have been kept at 20 °C and F1 offspring expressing the co-injection markers have been singled out. After the F1 offspring have laid embryos, they have been picked to 5 µl of single worm lysis buffer and the lysate has been used as PCR template to screen for mutant alleles. We isolated two deletion alleles of *Y45G5AM.2/pid-5* (*xf181* and *xf182*) and two deletion alleles of *W03G9.2/pid-4* (*xf184* and *xf185*). Each allele has been sequenced to pinpoint the exact deletion at nucleotide resolution. The mutant strains have been outcrossed two times against wild type N2 strain to remove any potential off-targets effect of Cas9 and used for further experiments.

Generation of endogenously tagged lines

In order to introduce an epitope tag at the endogenous loci of *W03G9.2/pid-4* and *Y45G5AM.2/pid-5*, we used the co-conversion approach as previously described (Arribere et al., 2014). Wild type worms have been injected with an injection mix containing 50 ng/µl pJS164 (Cas9 + sgRNA *dpy-10*); 750 nM ssODN SJ665 (repair oligo for *dpy-10*(*cn64*)); 50 ng/µl pRK1053; 750 nM ssODN SJ969 (repair oligo for W03G9.2::3xMyc) to introduce a 3xMyc epitope tag at the endogenous *W03G9.2/pid-4* locus (*xf186*). Wild type worms have been injected with an injection mix containing 50 ng/µl pJS164; 500 nM ssODN SJ665; 50 ng/µl pRK1060; 1000 nM ssODN SJ964 (repair oligo for Y45G5AM.2::2xHA) to introduce a 2xHA epitope tag at the endogenous *Y45G5AM.2/pid-5* locus (*xf192*). After injections, worms have been kept at 20 °C and F1 offspring with a roller phenotype (*rol-6*) have been singled out. After the F1 offspring have laid embryos, they have been picked to 5 µl of single worm lysis buffer and the lysate has been used as PCR template to screen for edited alleles. Each allele has been sequenced to ensure that the insertion of the epitope is in frame. The tagged strains have been outcrossed two times against wild type N2 strain to remove any potential off-targets effect of Cas9 and used for further experiments.

To introduce a fluorescent protein at the endogenous locus, we have first used a *unc-58* co-conversion approach (Arribere et al., 2014) to introduce at the *W03G9.2/pid-4* and at the *Y45G5AM.2/pid-5* loci a sequence of 20 nucleotides of *dpy-10* gene that serves as efficient protospacer sequence for subsequent edits (*xf204* and *xf221*, respectively), as previously described (Mouridi et al., 2017). We then used the generated strains (RFK932 and RFK972) as reference for the injection of a mix containing 50 ng/µl pJS164 (Cas9 + sgRNA *dpy-10*); 1000 nM SJ665 (repair oligo for *dpy-10*(*cn64*)); 300 ng/µl SJP010, a PCR product that was amplified from plasmid pDD286 (a gift from Bob Goldstein, Addgene plasmid # 70684) and used as a donor for the insertion of the mTagRFP-T sequence at the endogenous *W03G9.2/pid-4* and *Y45G5AM.2/pid-5* loci (Paix et al., 2014). After injections, worms have been kept at 20 °C and F1 offspring with a roller phenotype have been singled out. After the F1 offspring have laid embryos, they have been picked to 5 µl of single worm lysis buffer and the lysate has been used as PCR template to screen for edited alleles. We isolated the *xf206* and *xf226* alleles, which have been sequenced to ensure that the insertion of the mTagRFP-T sequence is in frame. The tagged strains have been outcrossed two times against wild type N2 strain to remove any potential off-targets effect of Cas9 and used for further experiments.

<u>List of primers and plasmids generated</u>

Plasmid	Oligo	Sequence
	p46169_RV	aaacatttagatttgcaatt
pRK1047	W03G9.2_sgRNA2	gcatgaacacgcataacgcgagttttagagctagaaatagc
pRK1050	W03G9.2_sgRNA5	gcaaagtacgcgaaagagggttttagagctagaaatagc
pRK1052	W03G9.2_sgRNA7	gcttcatatagctgccgctcggttttagagctagaaatagc
pRK1053	W03G9.2_sgRNA8	gaatatcaagcactagttggcgttttagagctagaaatagc
pRK1054	Y45G5AM.2_sgRNA1	ggctgtcatcagcgctcgtgttttagagctagaaatagc
pRK1056	Y45G5AM.2_sgRNA3	gtttcgcggttcgaagctacggttttagagctagaaatagc
pRK1057	Y45G5AM.2_sgRNA4	ggattggaatcaacgtgaggttttagagctagaaatagc
pRK1059	Y45G5AM.2_sgRNA6	gaatgagatcgatcgaagccggttttagagctagaaatagc
pRK1060	Y45G5AM.2_sgRNA7	caattaaaaatgctctagatgtttaagagctatgctggaaac

<u>List of ssODN repair templates</u>

SJ665

cacttgaacttcaatacggcaagatgagaatgactggaaaccgtaccgcatgcggtgcctatggtagcggagcttcacatggcttcagaccaacagcctat

SJ969
gatgaaaaataattaaagcttgaatatcaagcactacaagtcttcctcgctgatcaacttctgctcgaggtcctcctcggagatgagcttttgctcaagatcctcttcagaaataagtttttgttcacctccacctccggatccgttggccggcttcatatagctgccgctcgcgggaa

SJ964
cgaaaataacttaaaaacaattaaaaatgctctaggcatagtctggaacgtcatatgggtaagcgtaatctgggacatcgtatggataacctccacctccggatccgatcggctggcatgcattgaagagccagtcatattc

Protein extraction and immunoprecipitation using GFP-Trap®

Synchronized worms have been grown until adulthood, then washed with cold M9 buffer, collected in a final volume of 200 µl water and fast frozen on dry ice. 350 µl of 2X Lysis Buffer (20 mM Tris pH=7.5; 300 mM NaCl; 1 mM EDTA; 1% NP40; cOmplete™ Mini EDTA-free Protease Inhibitor Cocktail, Art. No. 11836170001, Roche) have been added to each sample. Worms have then been sonicated using Bioruptor Plus (30 seconds ON – 30 seconds OFF, 10 cycles, high) and lysates were spun down at maximum speed for 10 min at 4 °C. The cleared protein extracts (500 µl) have been transferred to a new tube and 30 µl of GFP-Trap®_M (Art. No. gtm-20, ChromoTek), previously equilibrated with Dilution/Wash Buffer (3 x 5 minutes) (10 mM Tris pH=7.5; 150 mM NaCl; 0,5 mM EDTA; cOmplete™ Mini EDTA-free Protease Inhibitor Cocktail), have been added to each sample and the immunoprecipitation was performed for 3 hours at 4 °C. Then, beads have been washed 3 x 5 minutes with 500 µl of Dilution/Wash Buffer. After the last wash, the beads have been resuspended in 25 µl of NuPAGE® LDS Sample Buffer 1x (Art. No. NP0007, Life technologies) with 100 mM DTT and boiled at 95 °C for 10 minutes.

Protein extraction and immunoprecipitation using Dynabeads™ Protein G

Synchronized worms have been grown until adulthood, then washed with cold M9 buffer, collected in a final volume of 200 µl water and fast frozen on dry ice. 350 µl of 2X Lysis Buffer (50 mM Tris HCl pH=7.5; 300 mM NaCl; 3 mM $MgCl_2$; 2 mM DTT; 0,2% Triton-X100; cOmplete™ Mini EDTA-free Protease Inhibitor Cocktail, Art. No. 11836170001, Roche) have been added to each sample. Worms have then been sonicated using Bioruptor Plus (30 seconds ON – 30 seconds OFF, 10 cycles, high) and lysates were spun down spun down at maximum speed for 10 min at 4 °C. The cleared protein extracts (500 µl) have been transferred to a new tube and 2 µg of antibody (αHA clone HA-7, Art. No. H3663, Sigma; αMYC 9B11, Art. No. 2276, Cell Signalling Technology; Monoclonal ANTI-FLAG® M2 antibody produced in mouse, Art. No. F3165, Sigma-Aldrich) or 1:100 of serum (823 αPID-2) have been added to each sample. The immunoprecipitation was performed for 2 hours at 4 °C. Then, 30 µl of Dynabeads™ Protein G (Art.No. 10004D, Invitrogen™), previously equilibrated with Wash Buffer (3 x 5 minutes) (25 mM Tris HCl pH=7.5; 150 mM NaCl; 1,5 mM $MgCl_2$; 1 mM DTT; cOmplete™ Mini EDTA-free Protease Inhibitor Cocktail), have been added and incubate for 1 additional hour at 4 °C. Then, beads have been washed 3 x 5 minutes with 500 µl of Wash Buffer. After the last wash, the beads have been resuspended in 25 µl of NuPAGE® LDS Sample Buffer 1x (Art. No. NP0007, Life technologies) with 100 mM DTT and boiled at 95 °C for 10 minutes.

Protein extraction and Western blot

For each strain, 50 L4 worms were hand-picked, to ensure comparable protein concentration and the right larval stage, in 13 µl of M9 buffer 1X. Subsequently, 5 µl of NuPAGE™ LDS Sample Buffer 4X (Art. No. NP0007, Invitrogen™) and 2 µl of DTT 1M have been added. Samples have been boiled at 95 ^{o}C for 10 minutes and then stored at -20 ^{o}C, until all samples have been collected.

Protein extracts have been loaded, together with a broad range protein ladder (Color Prestained Protein Standard, Broad Range; Art. No. # P7712; NEB), onto a 10% Bis-Tris gel (NuPAGE 10% Bis-Tris

Protein Gels, 1.0 mm, 10 well; Art. No. NP0301BOX; Invitrogen™) and run with MOPS buffer 1X (NuPAGE™ MOPS SDS Running Buffer (20X); Art. No. NP0001; Invitrogen™), at 140 V at 4 ^{o}C. Afterwards, proteins have been transferred on a PVDF membrane (Immobilon-P Membrane, PVDF, 0.45 µm; Art. No. IPVH00010; Merck Millipore) for 90 min, at 100 V at 4 ^{o}C, with Transfer buffer 1X (NuPAGE™ Transfer Buffer (20X); Art. No. NP00061; Invitrogen™) plus 10% methanol (Methanol, anhydrous 99.8%; Art. No. 322415-2L; Sigma-Aldrich Chemie GmbH). The membrane has been incubated with blocking buffer (5% milk in PBS-Tween 0,1%) for 1 h at room temperature. The membrane has then been cut, according to the molecular weight of the proteins to be detected, and each part has been incubated with a dilution of the primary antibody in 0,5% milk in PBS-Tween 0,1% (1:5000 Monoclonal ANTI-FLAG® M2 antibody produced in mouse, Art. No. F3165, Sigma-Aldrich; 1:5000 RFP Rabbit anti-Tag, Polyclonal, Art. No. 10041338, Fisher Scientific GmbH; 1:2500 Monoclonal anti-α-Tubulin antibody produced in mouse, Art. No. T6074, Sigma-Aldrich) for 1 h at room temperature. Then, three washes of five minutes each have been done with PBS-Tween 0,1% at room temperature. The three parts of the membrane have then been incubated with a dilution of the secondary antibody (1:10000 Anti-mouse IgG, HRP-linked Antibody, Art. No. #7076, Cell Signaling Technology; 1:10000 Anti-rabbit IgG, HRP-linked Antibody, Art. No. #7074, Cell Signaling Technology) in PBS-Tween 0,1% for 1 h at room temperature. Afterwards, three washes of five minutes each have been done with PBS-Tween 0,1% at room temperature, and the blot has been developed using the Amersham ECL Select Western Blotting Detection Reagent (Art. No. RPN2235, GE Healthcare) and detected at the ChemiDoc XRS+ (Bio-Rad).

2 Appendix Figures S1-S6

Figure S1

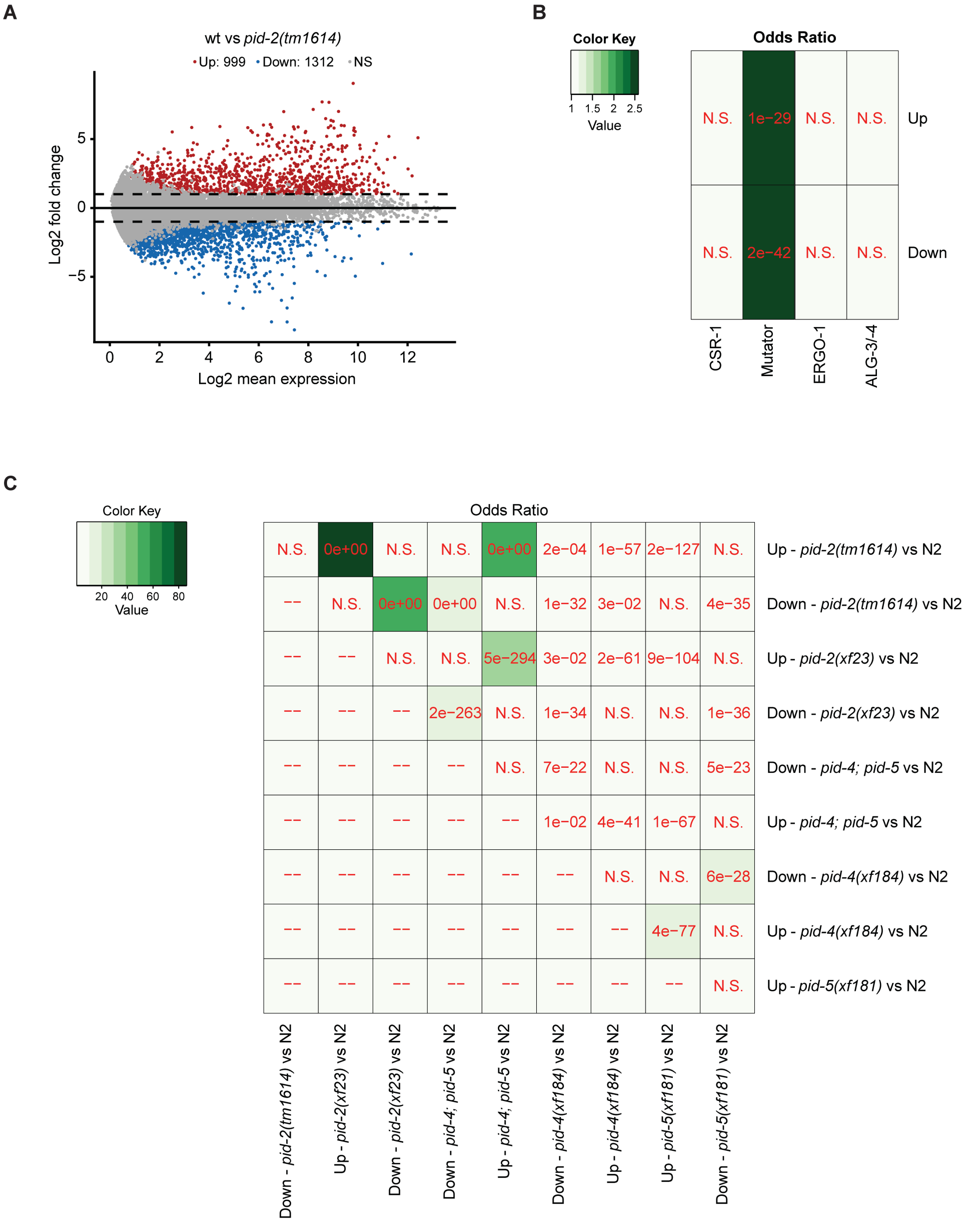

Appendix Fig S1. 22G analysis of *pid-2* mutants

A) MA-plot of $\log_2$ fold changes (Y-axis) versus the mean of normalized counts of 22G RNAs (X-axis) for *pid-2(tm1614)* mutants, compared to wild type. Red dots: genes with adjusted p-value < 0.05 and fold-change > 2. Blue dots: genes with adjusted p-value < 0.05 and fold-change < -2.

B) Heatmap displaying overlap significance between different gene sets and genes that are either up- or down-regulated in *pid-2(tm1614)* mutants. Significance was tested with Fisher's exact test and p-values adjusted with the Benjamini-Hochberg Procedure. Colour scheme represents the odds ratio of overlaps representing the strength of association. N.S.: not significant.

C) Heatmap displaying overlap significance between different gene sets and genes that either up- or down-regulated in the various mutants that are indicated. Significance was tested with Fisher's exact test and p-values adjusted with the Benjamini-Hochberg Procedure. Colour scheme represents the odds ratio of overlaps representing the strength of association. N.S.: not significant; --: ignored.

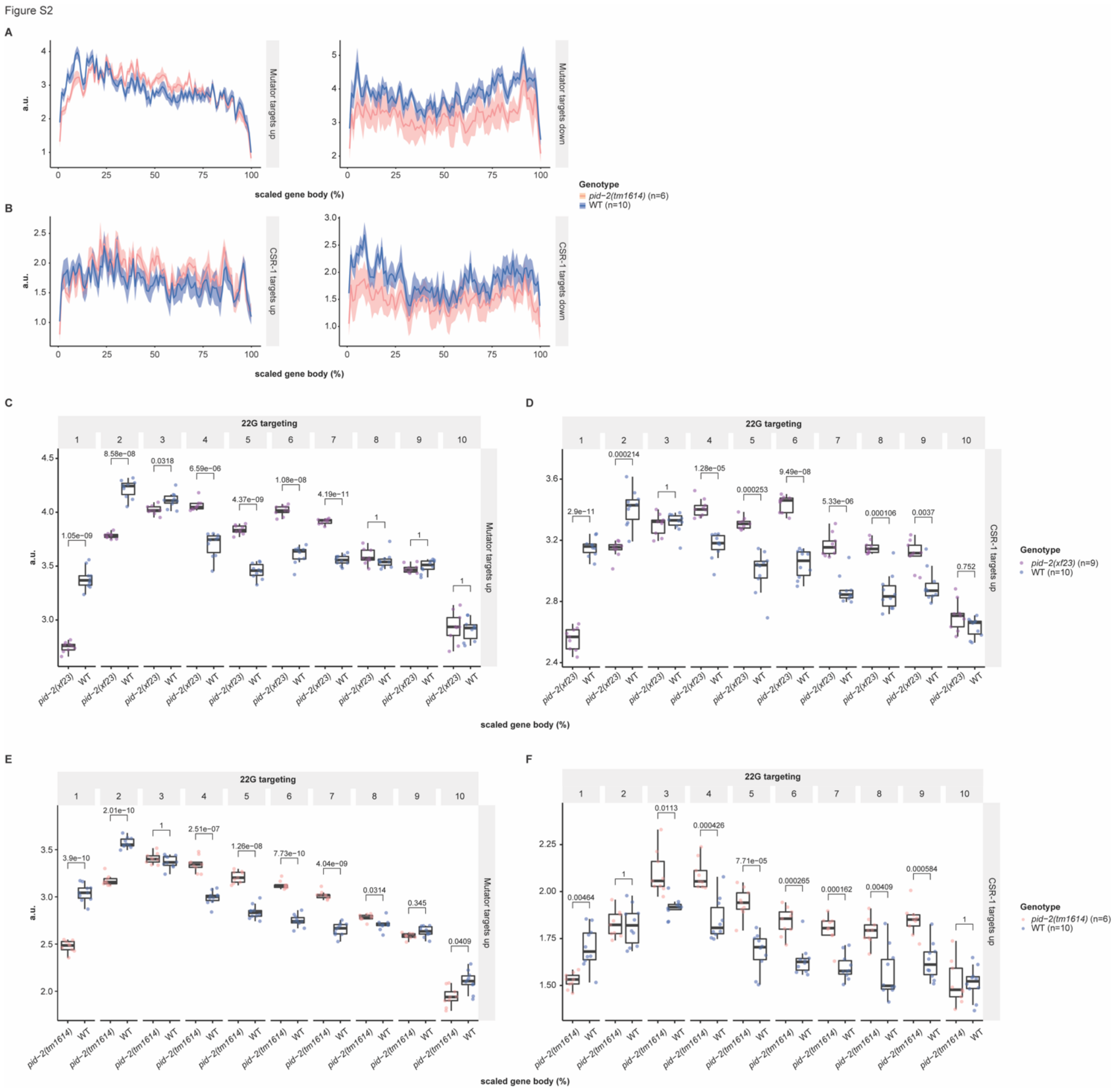

Appendix Fig S2. PID-2 affects 22G RNAs at the 5' ends of transcripts

A-B) 22G RNA coverage along the gene body of Mutator and CSR-1 targets. Values represent 22G coverage normalized to the total coverage of the gene, for wild type (N2) and *pid-2(tm1614)* mutants. Gene sets are previously defined 22G RNA target sub-types: CSR-1 (Conine et al., 2013), Mutator (Phillips et al., 2014). The lines represent the average of biological replicates, whereas the shading represents the standard deviation of biological replicates. a.u.: arbitrary units.

C-F) Cumulative 22G coverage, binned into 10% gene-length bins, along the gene body of Mutator and CSR-1 targets. Values represent 22G coverage normalized to the total coverage of the gene, for wild type (N2) and *pid-2(xf23)* (C, D) and *pid-2(tm1614)* (E, F) mutants. The presented data is the same as that in Figure 3 C and D, and S2A and B respectively, but coverage was binned to assess statistical significance. p-values were calculated with unpaired t-test and corrected for multiple testing with the Bonferroni method. Gene sets are previously defined 22G RNA target sub-types (See Appendix). a.u.: arbitrary units.

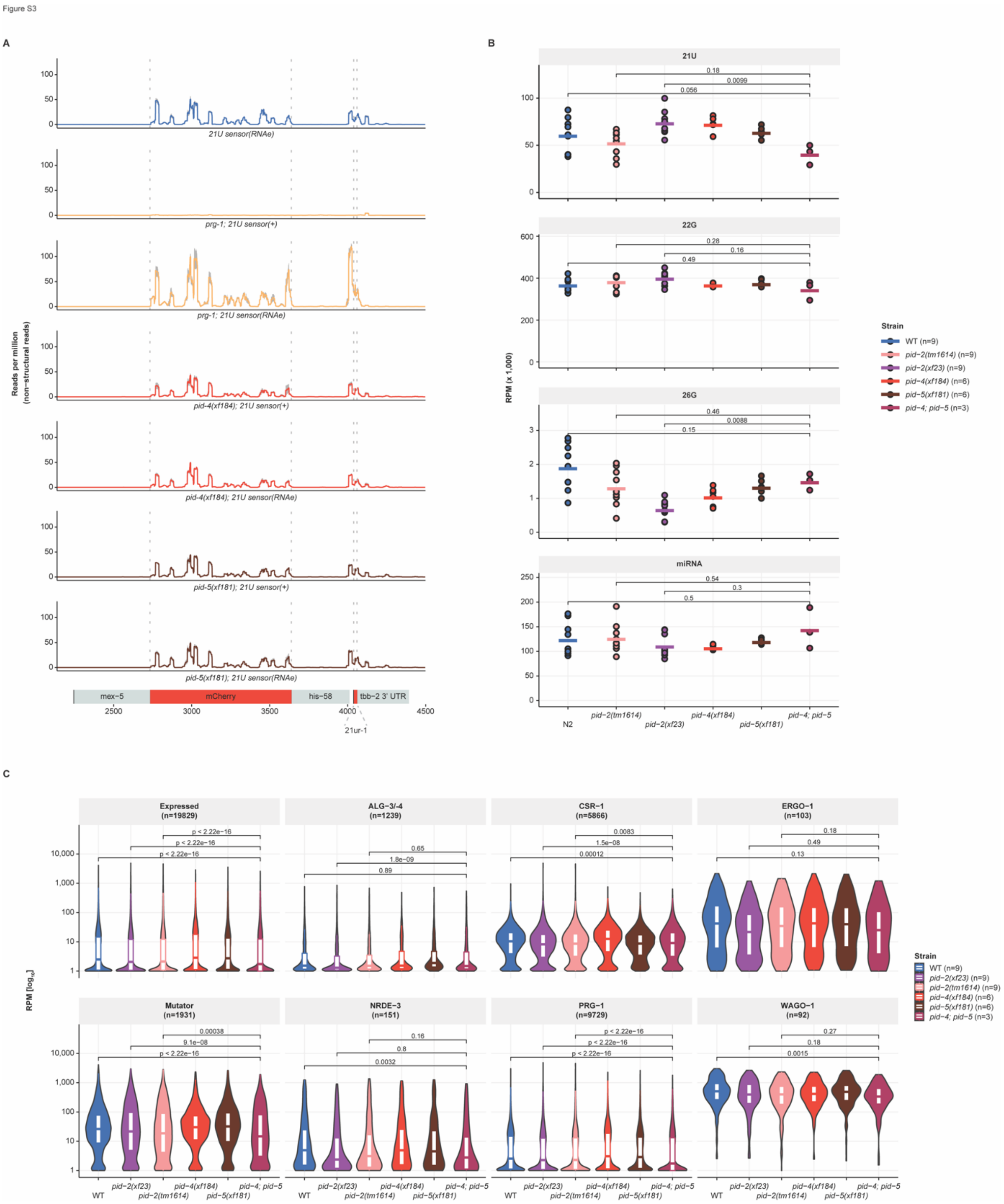

Appendix Fig S3. 22G RNA analysis of *pid-4* and *pid-5* mutants

A) Plots representing 22G RNA reads mapping against the 21U sensor, schematically represented at the bottom, in wild-type, *pid-4* and *pid-5* mutant backgrounds. The 21U sensor was crossed into the two mutant backgrounds in either a silenced state, from a *prg-1* mutant (RNAe), or in an active state, from a *mut-7* mutant (indicated as +). Both *mut-7* and *prg-1* were confirmed to be wild type in the strains that were isolated from these crosses. In each plot, the average of three biological replicates is represented and the shading represents the standard deviation among the replicates.

B) Representation of the total abundance of small RNA classes (21U, 22G, 26G RNAs and miRNAs) from small RNA sequencing of the indicated genetic backgrounds. Each replicate is represented by a dot and

the median is represented by a bar. P-values are calculated with a two-tailed unpaired t-test. RPM: reads per million.

C) Violin plots representing the distribution of different sub-types of 22G RNAs as previously defined, as a group, in the indicated genetic backgrounds. The white boxes inside each of the violin plots represent the 75th and 25th percentile of the distribution, top and bottom respectively. The median of the distribution is represented by the line in each box. P-values are calculated with a two-sided unpaired Mann-Whitney/Wilcoxon rank-sum test, indicating the differences between *pid-2* mutants and either wild type or *prg-1* mutants as references. RPM: reads per million.

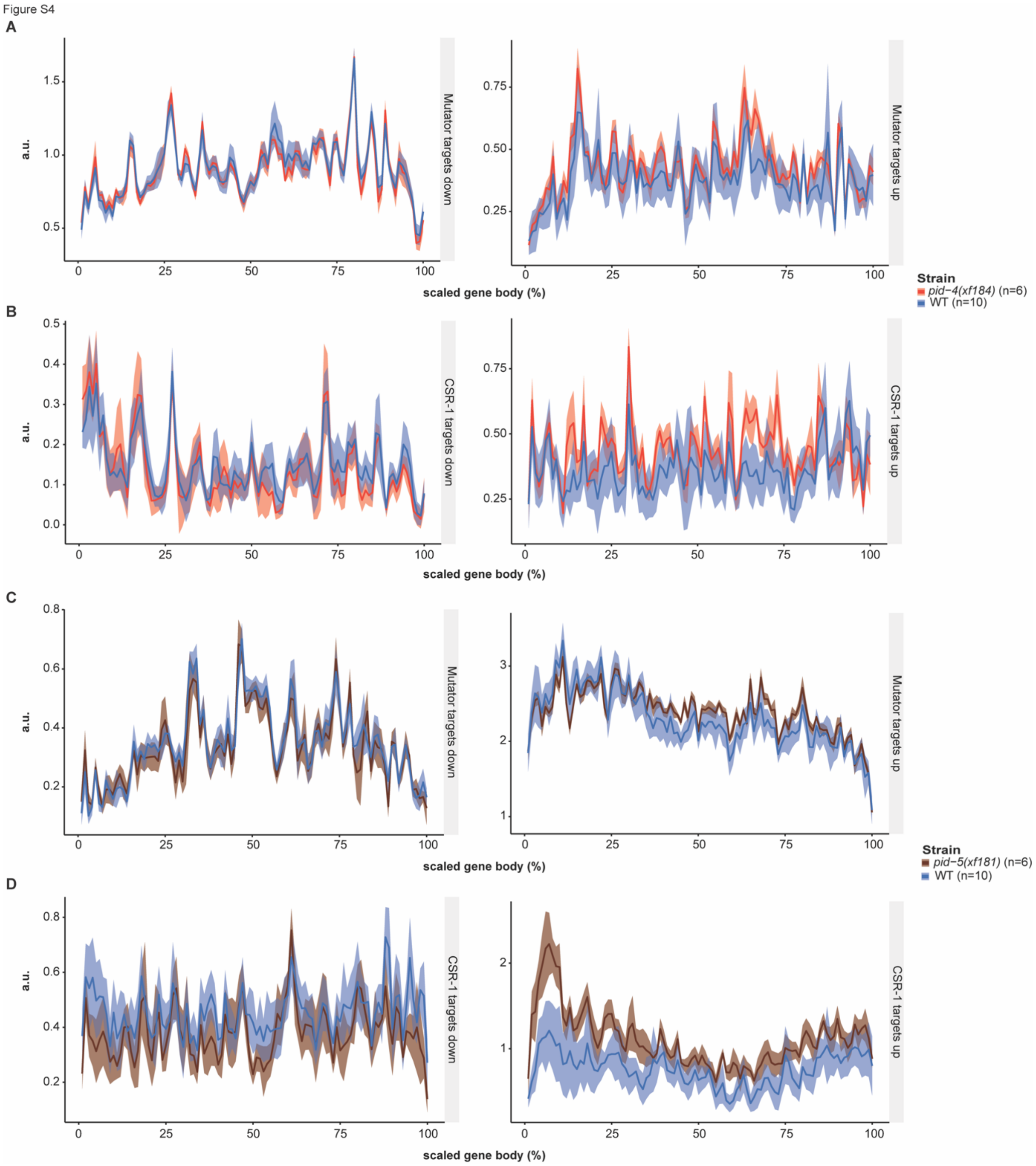

Appendix Fig S4. Metagene analysis of mutator and CSR-1 targets in *pid-4* and *pid-5* mutants

A-D) Cumulative 22G coverage along the gene body of CSR-1 and Mutator targets. Values represent 22G coverage normalized to the total coverage of the gene, for wild type (N2) and *pid-4* (A-B) or *pid-5* (C-D) mutants. The lines represent the average of biological replicates, whereas the shading represents the standard deviation of biological replicates. a.u.: arbitrary units.

Figure S5

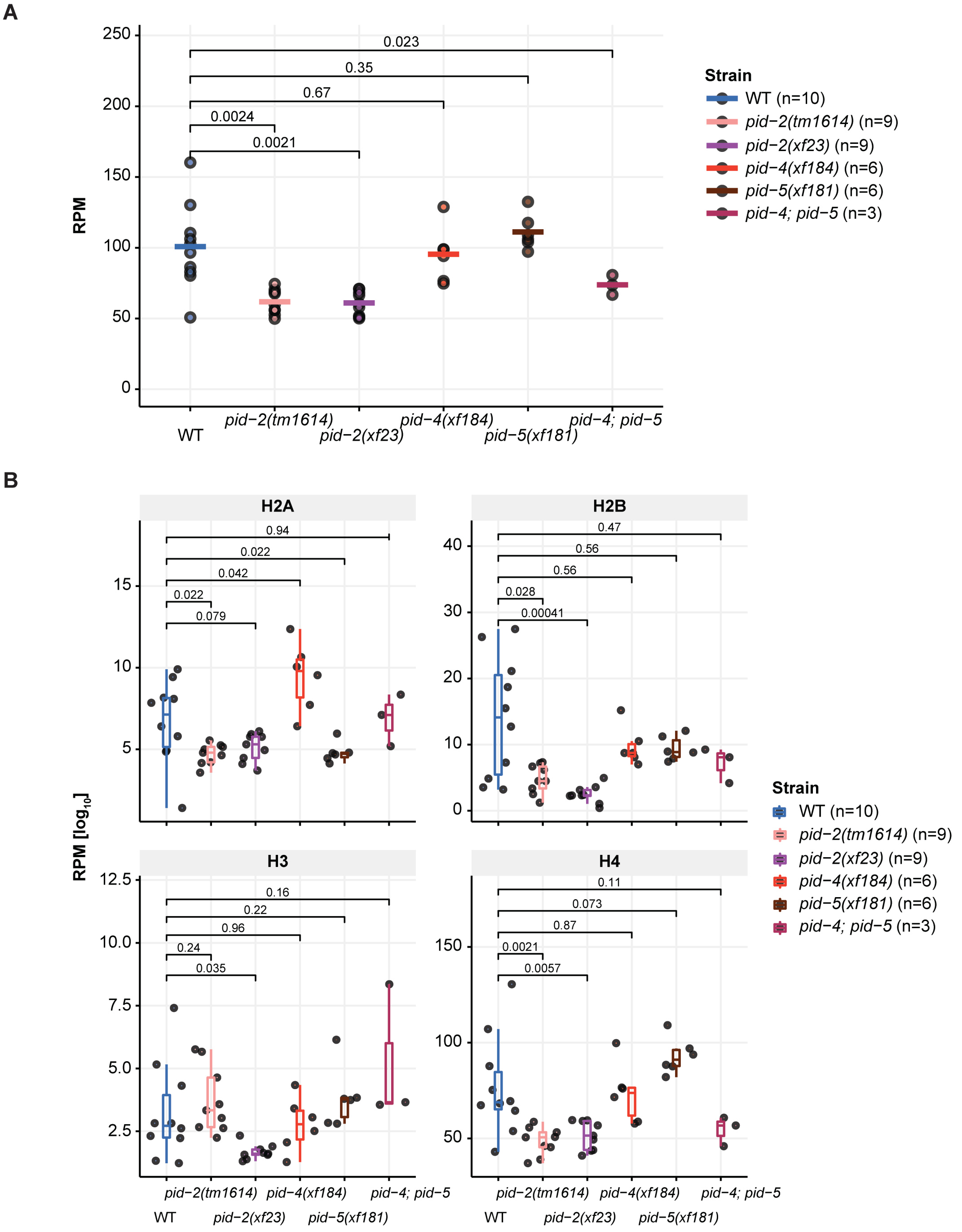

Appendix Fig S5. Histone derived 22G RNAs do not increase in *pid-2*, *pid-4* and *pid-5* mutants

A) Histone-derived 22G RNAs detected in the indicated mutant backgrounds. Reads from all replication-dependent histones were merged for this plot. Each replicate is represented by a dot and the median is represented by a bar. P-values are calculated with a two-tailed unpaired t-test. RPM: reads per million.

B) Histone-derived 22G RNAs detected in the indicated mutant backgrounds. Reads from the different replication-dependent histones were split for this plot. (See supplemental methods for details). Each replicate is represented by a dot and the median is represented by a bar. P-values are calculated with a two-tailed unpaired t-test. RPM: reads per million.

Figure S6

A

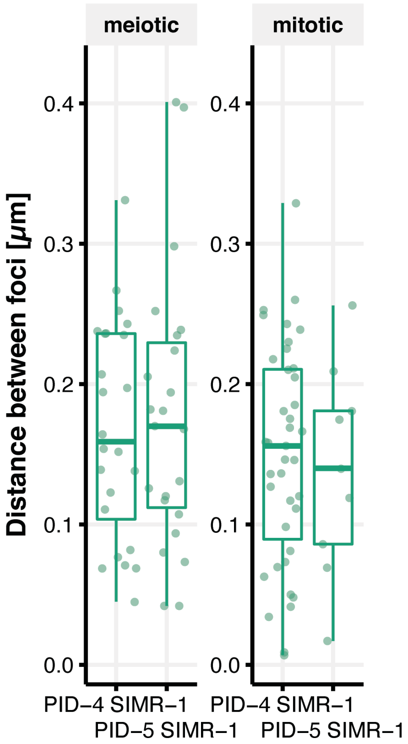

B

PID-4::mTagRFP-T (p-value = 0.014)		
	**wild type**	***pid-2(xf23)***
**Total nuclei**	23	26
**Total foci**	169	122
**Average number foci / nucleus**	7.3	4.7

PID-5::mTagRFP-T (p-value = 0.36)		
	**wild type**	***pid-2(xf23)***
**Total nuclei**	24	25
**Total foci**	80	69
**Average number foci / nucleus**	3.3	2.8

C

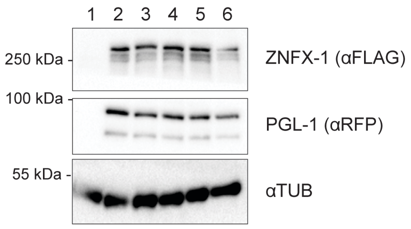

1: N2
2: *znfx-1(gg544[3xflag::gfp::znfx-1]); pgl-1(xf233[pgl-1::mTagRfp-t])*
3: *znfx-1(gg544[3xflag::gfp::znfx-1]); pgl-1(xf233[pgl-1::mTagRfp-t]); pid-2(xf23)*
4: *znfx-1(gg544[3xflag::gfp::znfx-1]); pgl-1(xf233[pgl-1::mTagRfp-t]); pid-4; pid-5*
5: *znfx-1(gg544[3xflag::gfp::znfx-1]); pgl-1(xf233[pgl-1::mTagRfp-t]); pid-4(xf184)*
6: *znfx-1(gg544[3xflag::gfp::znfx-1]); pgl-1(xf233[pgl-1::mTagRfp-t]); pid-5(xf181)*

Appendix Fig S6. Colocalization analysis

A) Box-plots representing the distance (µm) between the centres of two fluorescent signals from the indicated fusion proteins, as represented in Figure EV5B. The distance between each pair of closest fluorescent signals is represented by a dot. The median is represented by a line. The 75th and 25th percentile are represented by the upper and lower lines, respectively. P-values were calculated using an unpaired t-test (two-tailed).

B) Tables listing the number of PID-4::mTagRFP-T (**D**) and PID-5::mTagRFP-T (**E**) foci and the number of nuclei used for quantification in a wild-type and in a *pid-2* mutant background, represented in Figure 6D, E and 7G, H. For each cropped area, the number of nuclei and foci was counted, and statistical differences between wild type and *pid-2* mutants were determined with a one-tailed weighted Student's t-test, implemented in the R package "weights", which accounts for the different number of nuclei in each area. P-values are indicated in the tables.

C) Western blot to detect the expression of 3xFLAG::GFP::ZNFX-1, using αFLAG antibody, and PGL-1::mTagRFP-T, using αRFP antibody, in the indicated strains. Per lane, 50 L4 larvae were used to make a lysate by boiling in NuPAGE® LDS sample buffer. αTubulin has been used as loading control.
