# Supplementary material for: Intrinsically disordered protein PID‐2 modulates Z granules and is required for heritable piRNA‐induced silencing in the Caenorhabditis elegans embryo
Source: EMBO J. 2020 Nov 24;40(3):e105280. doi: 10.15252/embj.2020105280 (PMC7849312; doi:10.15252/embj.2020105280)
Supplement: Supplementary file 2 — Expanded View Figures PDF [file EMBJ-40-e105280-s002.pdf]

Expanded View Figures

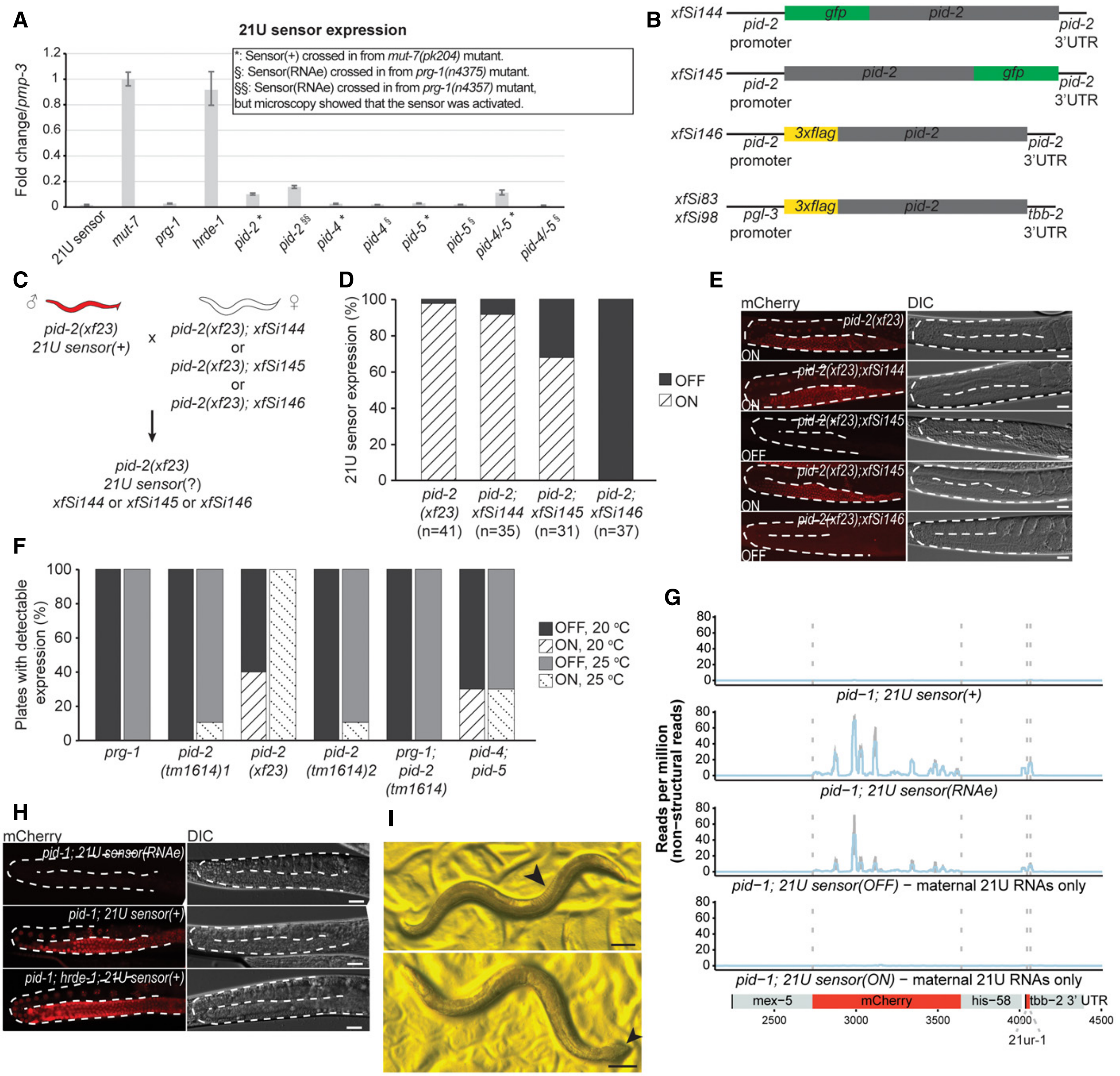

Figure EV1.

 © 2020 The Authors

Figure EV1. PID-2 affects 21U sensor expression.

A RT-qPCR of the 21U sensor in the indicated mutant backgrounds. *: strains that received the 21U sensor from a *mut-7* background (active). §: strains that received a silenced 21U sensor from a *prg-1* background (RNAe). Expression of *pmp-3* was used to normalize the data. Error bars reflect the standard deviation, calculated from three technical replicates. We note that the *pid-2* strain, that received the 21U sensor originally in an RNAe state, has RNA levels of the 21U sensor comparable to a de-silenced sensor in a *pid-2* mutant background. Indeed, upon re-examination of this strain under the microscope, we detected expression of the 21U sensor, indicating that in this isolate RNAe had been lost (also see Fig EV1F). Bars represent the fold change of the 21U sensor expression compared with the control (*pmp-3*). Error bars represent mean ± SD. For each strain, three replicates have been used.

B Scheme of the single-copy MosSCI and miniMos transgenes expressing tagged PID-2. The MosSCI transgenes (*xfSi144, xfSi145, xfSi146*) are driven by the endogenous promoter and 3′ UTR and were inserted into a known germline-expressing site on chromosome II, *ttTi5605*. The miniMos transgenes (*xfSi83*, *xfSi98*) instead are driven by a germline-specific promoter (*pgl-3*) and 3′UTR (*tbb-2*) and have been randomly inserted in the genome. The *xfSi83* transgene was mapped to chromosome II, within the last intron of *mpz-1*, whereas the transgene *xfSi98* was mapped to chromosome V, within the fourth intron of the Y32B12B.4 gene.

C Crossing strategy to assess the rescue of the *pid-2* mutation by the indicated *pid-2* transgenes.

D Quantification of the expression of the 21U sensor (% of animals analysed) in the indicated mutant backgrounds as a measure of the rescue of the *pid-2* mutation by the different *pid-2* transgenes. Expression was quantified by scoring individuals (ON/OFF) using microscopy.

E Representative images of the 21U sensor expression (left: mCherry signal; right: DIC) in the indicated genetic backgrounds. For *xfSi145*, both ON and OFF states are depicted. Gonads are outlined by a dashed line. Scale bar: 25 μm.

F Quantification of the reactivation of the 21U sensor (RNAe) (indicated as % of plates containing animals with detectable expression), at either 20°C or at 25°C, in the indicated mutant backgrounds. From each of the indicated mutant backgrounds, 20 L2-L3 larvae were singled out and scored to ensure that the 21U sensor was still silenced (RNAe). Then, 10 plates were kept at 20°C and 10 plates were kept at 25°C and chunked regularly to avoid starvation. After 14 days, plates were scored by microscopy for reactivation of the 21U sensor (RNAe). When expression was detected on a plate, the majority of animals showed expression (~60–80%). *pid-2 (tm1614)*$_1$ and *pid-2(tm1614)*$_2$ represent two independently generated strains with the same genotype (RFK530 and RFK586, respectively).

G 22G RNA profile on the 21U sensor schematically represented at the bottom, in the indicated mutant backgrounds. The top two panels refer to control strains, whereas the bottom two panels show profiles from strains isolated from an individual in which the 21U sensor was exposed to maternal 21U RNAs only. Both silenced and non-silenced strains were sequenced. In each plot, the average of three biological replicates is represented. The shading represents the standard deviation among the replicates.

H Representative images of the 21U sensor expression (left: mCherry signal; right: DIC) in the indicated genetic backgrounds. The two top panels represent strains that have been exposed to maternal 21U RNAs only. In the strain depicted on top, the sensor became silenced, whereas in the strain depicted below it did not get silenced. Upon introduction of *hrde-1* mutation in the strain carrying a 21U sensor silenced upon exposure to maternal 21U RNAs only, the 21U sensor is reactivated, as shown at the bottom. Gonads are outlined by a dashed line. Scale bar: 25 μm.

I Examples of *pid-1;pid-2* double mutant animals, isolated from a growing *pid-1;pid-2* double mutant population, showing feminization (upper panel) and pseudo-males (lower panel). The arrow indicates arrayed appearance of oocytes in the feminized animal (upper panel), while it indicates a male-like tail in the pseudo-male (lower panel). The latter also shows characteristics of hermaphrodites (two gonad arms, eggs in uterus and vulva). Scale bar: 100 μm.

Source data are available online for this figure.

© 2020 The Authors

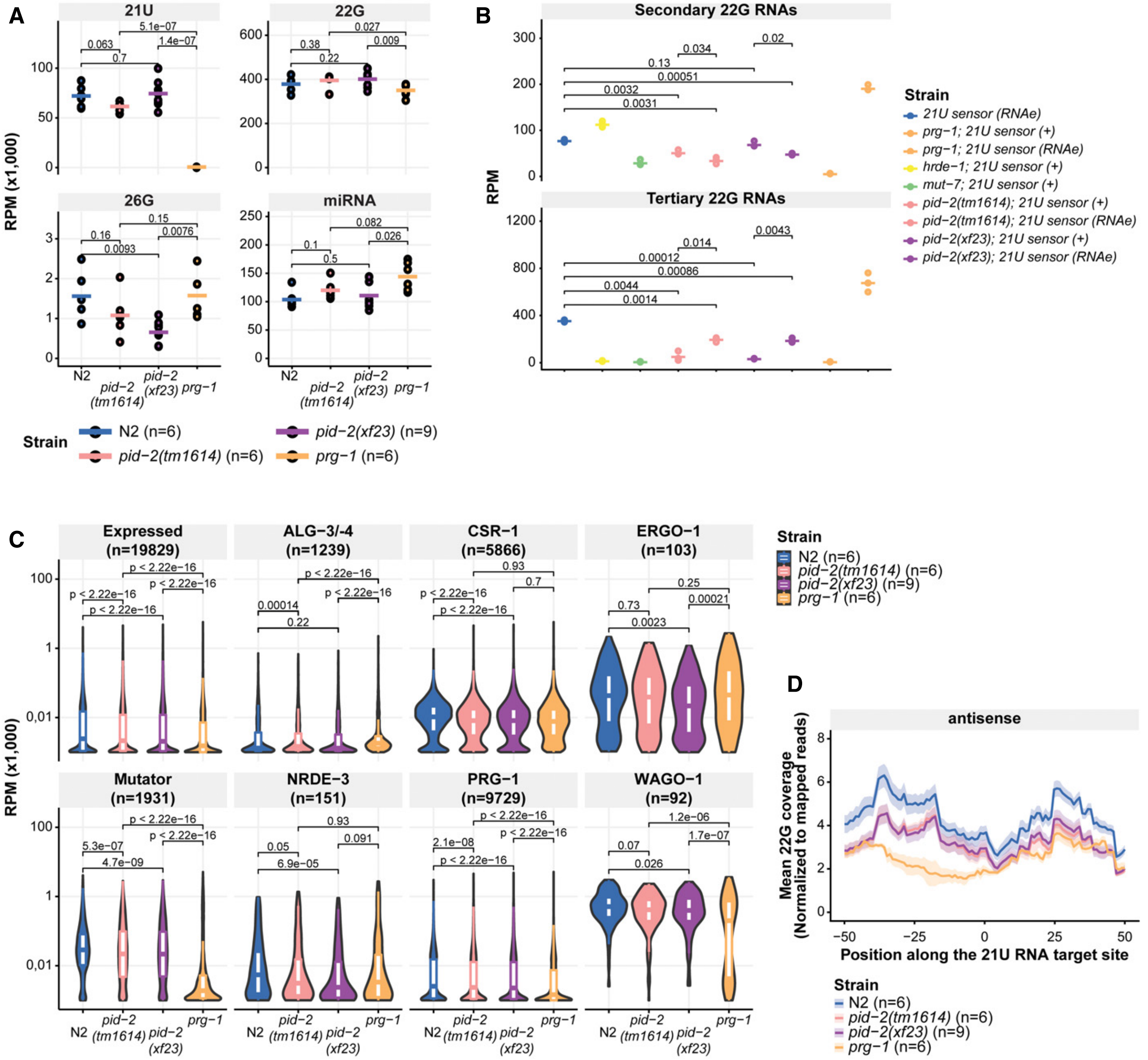

Figure EV2. PID-2 affects 22G RNAs.

A Representation of the total abundance of small RNA classes (21U, 22G, 26G RNAs and miRNAs) from small RNA sequencing data of the indicated genetic backgrounds. Each replicate is represented by a dot, and the median is represented by a bar. *P*-values are calculated with a two-tailed unpaired *t*-test. RPM: reads per million. N2: wild type.

B Dot plot for quantification of secondary (around the 21U RNA recognition site; upper panel) and tertiary (within the mCherry coding region; lower panel) 22G RNAs complementary to the 21U sensor in the indicated genetic backgrounds. Each replicate is represented by a dot, and the median is represented by a bar. *P*-values are calculated with a two-tailed unpaired t-test. All genotypes had 3 replicates. RPM: reads per million.

C Violin plots representing the distribution of different sub-types of 22G RNAs as previously defined, as a group, in the indicated genetic backgrounds. The white boxes inside each of the violin plots represent the 75th and 25th percentile of the distribution, top and bottom, respectively. The median of the distribution is represented by the line in each box. *P*-values are calculated with a two-sided unpaired Mann–Whitney/Wilcoxon rank-sum test, indicating the differences between *pid-2* mutants and either wild type or *prg-1* mutants as references. RPM: reads per million.

D Profile of antisense 22G RNAs produced in a 100-bp window around endogenous 21U RNA target sites (+50 bp; −50 bp) of WAGO-1 target genes, centred on position 10 of the 21U RNA sequence. Each line represents a specific genotype, and the shading represents the standard deviation of the biological triplicates for the indicated genetic backgrounds.

 © 2020 The Authors

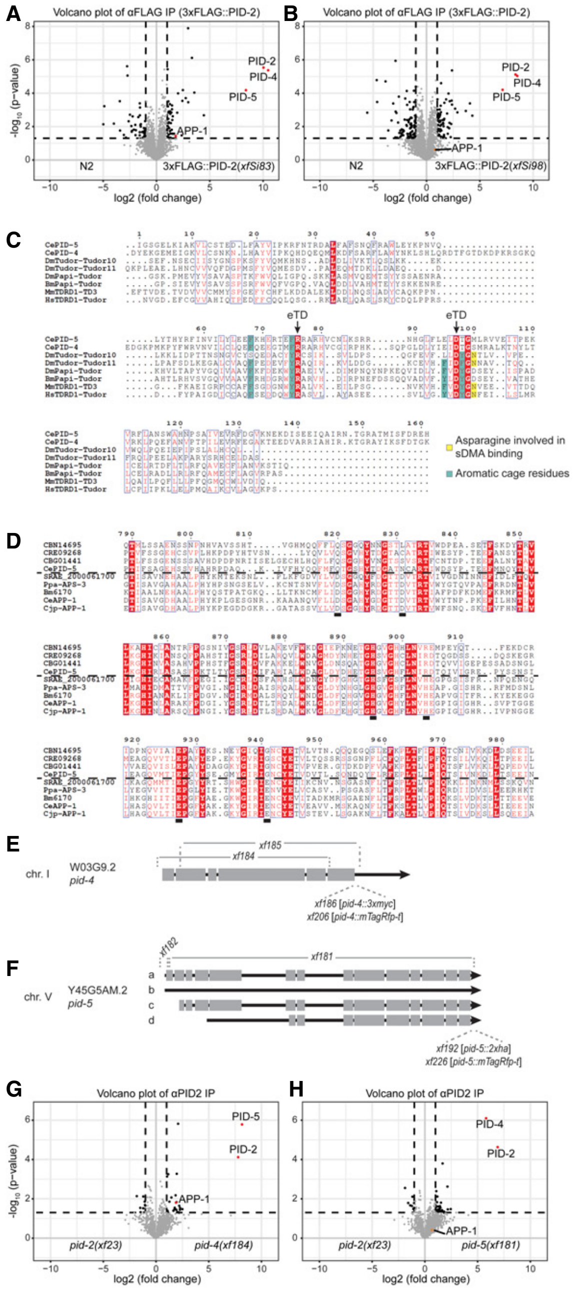
A
Volcano plot of αFLAG IP (3xFLAG::PID-2)
PID-2
PID-4
PID-5
APP-1
N2
3xFLAG::PID-2(xfSi83)
-log10 (p-value)
log2 (fold change)
B
Volcano plot of αFLAG IP (3xFLAG::PID-2)
PID-2
PID-4
PID-5
APP-1
N2
3xFLAG::PID-2(xfSi98)
C
CePID-5
CePID-4
DmTudor-Tudor10
DmTudor-Tudor11
DmPapi-Tudor
BmPapi-Tudor
MmTDRD1-TD3
HsTDRD1-Tudor
eTD
Asparagine involved in sDMA binding
Aromatic cage residues
D
CBN14695
CRE09268
CBG01441
CePID-5
SRAE_2000061700
Ppa-APS-3
Bm6170
CeAPP-1
Cjp-APP-1
E
chr. I
W03G9.2
pid-4
xf185
xf184
xf186 [pid-4::3xmyc]
xf206 [pid-4::mTagRfp-t]
F
chr. V
Y45G5AM.2
pid-5
xf182
xf181
a
b
c
d
xf192 [pid-5::2xha]
xf226 [pid-5::mTagRfp-t]
G
Volcano plot of αPID2 IP
PID-5
PID-2
APP-1
pid-2(xf23)
pid-4(xf184)
H
Volcano plot of αPID2 IP
PID-4
PID-2
APP-1
pid-2(xf23)
pid-5(xf181)

Figure EV3.

◀

Figure EV3. PID-2 interacts with PID-4 and PID-5.

A, B Volcano plot representing the enrichment of proteins interacting with PID-2, as determined by immunoprecipitation of 3xFLAG::PID-2 protein, followed by quantitative label-free mass spectrometry. Results from two strains with independently generated miniMos transgenes (*xfSi83* in A and *xfSi98* in B) are shown. As control, FLAG IPs were performed on extracts from wild-type, non-transgenic animals. For each IP-MS experiment, quadruplicates were measured and analysed. The dashed line reflects a significance threshold of P-value < 0.05 at twofold enrichment.

C Multiple sequence alignment, using ESPript 3 (http://espript.ibcp.fr) (Robert & Gouet, 2014), of the predicted eTudor domains of PID-5 and PID-4 with eTudor domains of proteins from other organisms. If the conservation is > 70%, the columns are framed in blue. The residues perfectly conserved are highlighted in red. The residues written in black are not conserved. Dots (.) indicate gaps in the protein sequence generated through the alignment. The residue characteristics of eTudor domains are indicated by the arrow (eTD). The aromatic residues forming the aromatic cage are highlighted in green, and the conserved acidic amino acid residue is highlighted in yellow. Ce: *Caenorhabditis elegans*; Dm: *Drosophila melanogaster*; Bm: *Bombyx mori*; Mm: *Mus musculus*; Hs: *Homo sapiens*.

D Multiple sequence alignment of the X-Prolyl aminopeptidase domain of PID-5 (CePID-5) with its orthologs, and with APP-1 (CeAPP-1) and APP-1 orthologs from other nematodes, using ESPript 3 (http://espript.ibcp.fr) (Robert & Gouet, 2014). Conservation > 70% is framed in blue. Perfectly conserved residues are highlighted in red. The residues in black are not conserved. Dots indicate gaps in the protein sequence generated through the alignment. The dashed line separates the protein according to the presence or absence of the catalytic residues, which are underlined. *C. brenneri*: CBN14695; *C. remanei*: CRE09268; *C. briggsae*: CBG01441; *S. ratti*: SRAE_2000061700; *P. pacificus*: Ppa-APS-3 PPA08577; *B. malayi*: Bm6170; *C. japonica*: Cjp-APP-1.

E Genomic locus and predicted transcript (Wormbase) for *pid-4*. The alleles generated using CRISPR/Cas9 technology are indicated. *xf184*: −1570 +8 nt; *xf185*: −1587 nt; *xf186*: 3xMyc; *xf206*: mTagRFP-T.

F Genomic locus and predicted transcripts (Wormbase) for *pid-5*. The alleles generated using CRISPR/Cas9 technology are indicated. *xf181*: −5291 +5 nt; *xf182*: −114 +3 nt; *xf192*: 2xHA; *xf226*: mTagRFP-T.

G, H Volcano plots representing the enrichment of proteins interacting with PID-2, as determined by immunoprecipitation of the endogenous PID-2 protein, followed by quantitative label-free mass spectrometry. IPs on protein extracts from *pid-2* mutant were compared to IPs on protein extracts from *pid-4* (G) and *pid-5* (H) mutants. For each IP-MS experiment, quadruplicates were measured and analysed. The dashed line reflects a significance threshold of P-value < 0.05 at twofold enrichment.

© 2020 The Authors

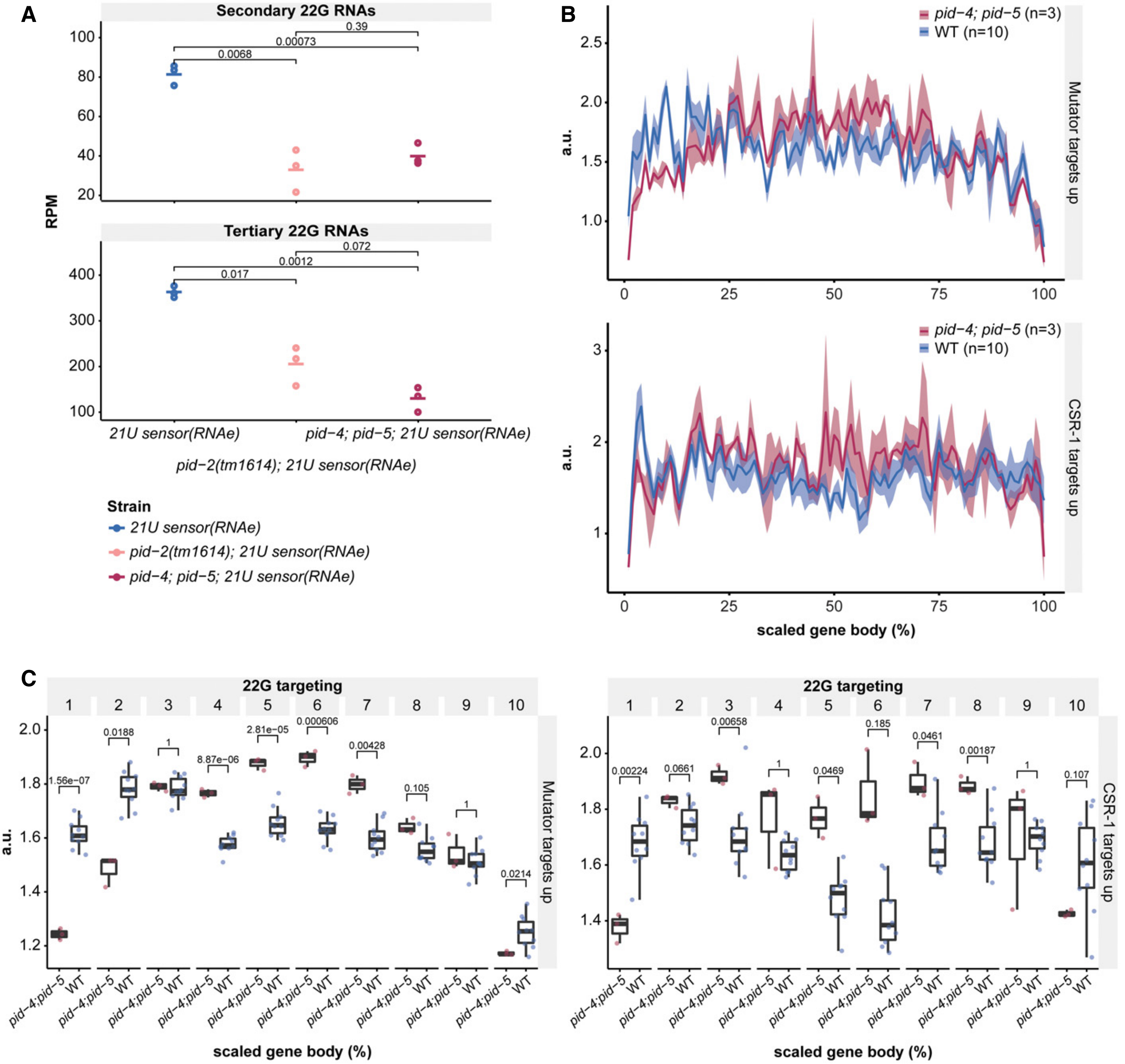

Figure EV4. PID-4 and PID-5 affect 22G RNAs.

A Dot plot for quantification of secondary (around the 21U RNA recognition site; upper panel) and tertiary (within the coding region; lower panel) 22G RNAs complementary to the 21U sensor (RNAe) in the indicated genetic backgrounds. Each replicate is represented by a dot, and the median is represented by a bar. *P*-values are calculated with a two-tailed unpaired *t*-test. RPM: reads per million.

B Cumulative 22G coverage along the gene body of Mutator and CSR-1 targets. Values represent 22G coverage normalized to the total coverage of each gene, for wild type (N2) and *pid-4;pid-5* double mutants. Gene sets are previously defined 22G RNA target sub-types (see Appendix). The lines represent the average of biological replicates, whereas the shading represents the standard deviation of biological replicates. a.u.: arbitrary units.

C Cumulative 22G coverage, binned into 10% gene-length bins, along the gene body of Mutator and CSR-1 targets. Values represent 22G coverage normalized to the total coverage of the gene, for wild type (N2) and *pid-4;pid-5* double mutants. The presented data are the same as that in panel B, but coverage was binned to assess statistical significance. *P*-values were calculated with a two-tailed unpaired *t*-test and corrected for multiple testing with the Bonferroni method. Gene sets are previously defined 22G RNA target sub-types (See Appendix). The median is represented by a line. The interquartile range (IQR), 25th to 75th percentile, is represented by the upper and lower lines, respectively, and whiskers represent the first quartile (down to −1.5*IQR) or the third quartile (up to +1.5*IQR). a.u.: arbitrary units.

© 2020 The Authors

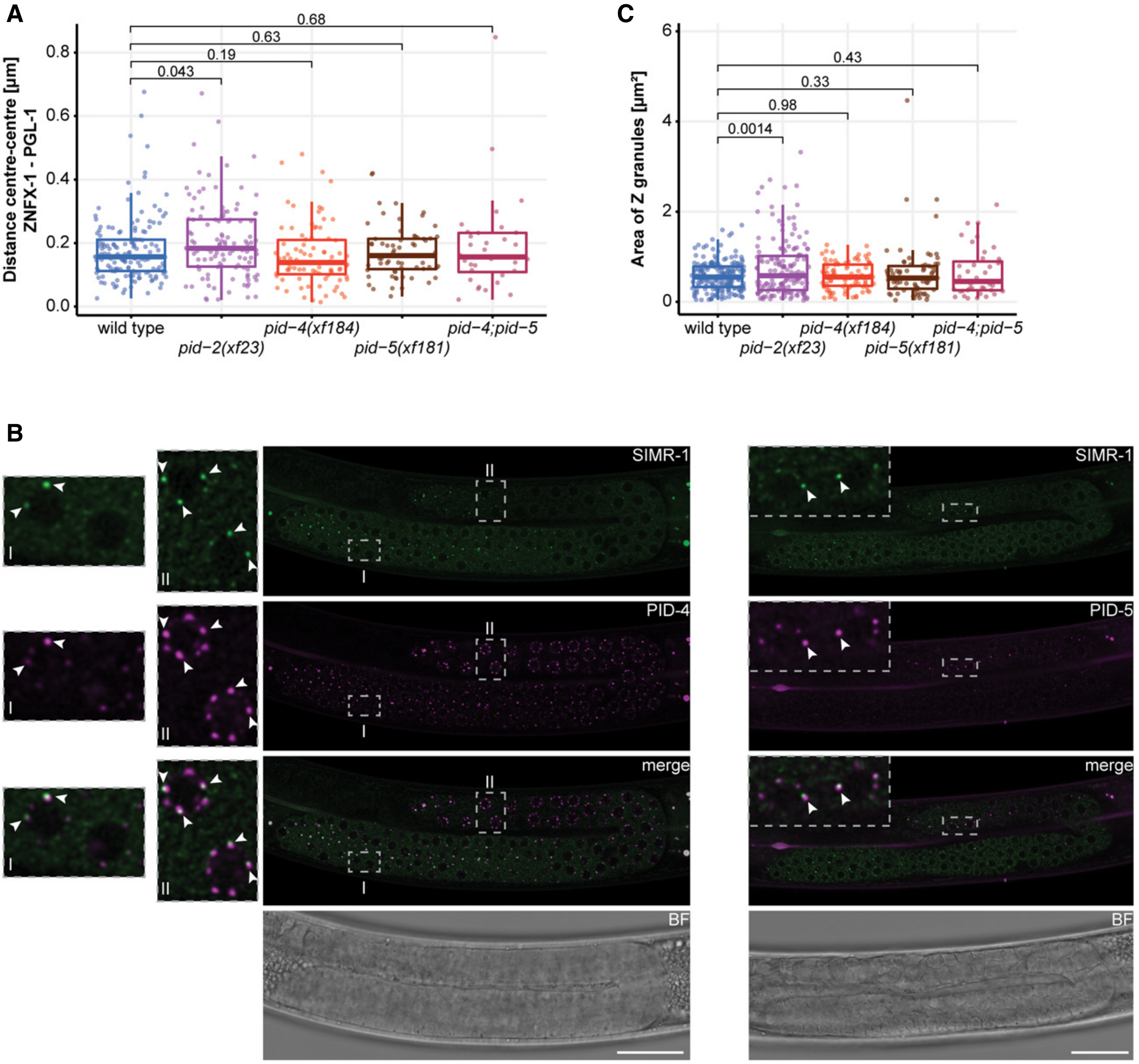

Figure EV5. Colocalization analysis with different germ granule markers.

A Box plots representing the distance (µm) between P and Z granules, in wild type, *pid-2*, *pid-4;pid-5*, *pid-4* and *pid-5* mutant backgrounds. The distance between each pair of fluorescent proteins is represented by a dot. Between 4 and 10 different gonads were analysed for each condition. The median is represented by a line. The interquartile range (IQR), 25th to 75th percentile, is represented by the upper and lower lines, respectively, and whiskers represent the first quartile (down to −1.5*IQR) or the third quartile (up to +1.5*IQR). *P*-values were calculated using an unpaired *t*-test (two-tailed).

B Expression of PID-4::mTagRFP-T (left) and PID-5::mTagRFP-T (right) together with SIMR-1::GFP. The indicated dashed boxes reflect zoom-ins on specific nuclei to better visualize the granules, and their overlaps. One L4 gonad is shown for each animal. Note that most of the L4 gonad is in pachytene stage. Arrowheads indicate individual condensates. Scale bar: 25 µm.

C Box plots representing the area of Z granules (μm^2) in wild type, *pid-2*, *pid-4;pid-5*, *pid-4* and *pid-5* mutant backgrounds. The area of each Z granule is represented by a dot. Between 4 and 10 different gonads were analysed for each condition. The median is represented by a line. The interquartile range (IQR), 25th to 75th percentile, is represented by the upper and lower lines, respectively, and whiskers represent the first quartile (down to −1.5*IQR) or the third quartile (up to +1.5*IQR). *P*-values were calculated using an unpaired *t*-test (two-tailed).

Source data are available online for this figure.

© 2020 The Authors
