# Supplementary figures and images for: Intrinsically disordered protein PID‐2 modulates Z granules and is required for heritable piRNA‐induced silencing in the Caenorhabditis elegans embryo
Source: EMBO J. 2020 Nov 24;40(3):e105280. doi: 10.15252/embj.2020105280 (PMC7849312; doi:10.15252/embj.2020105280)

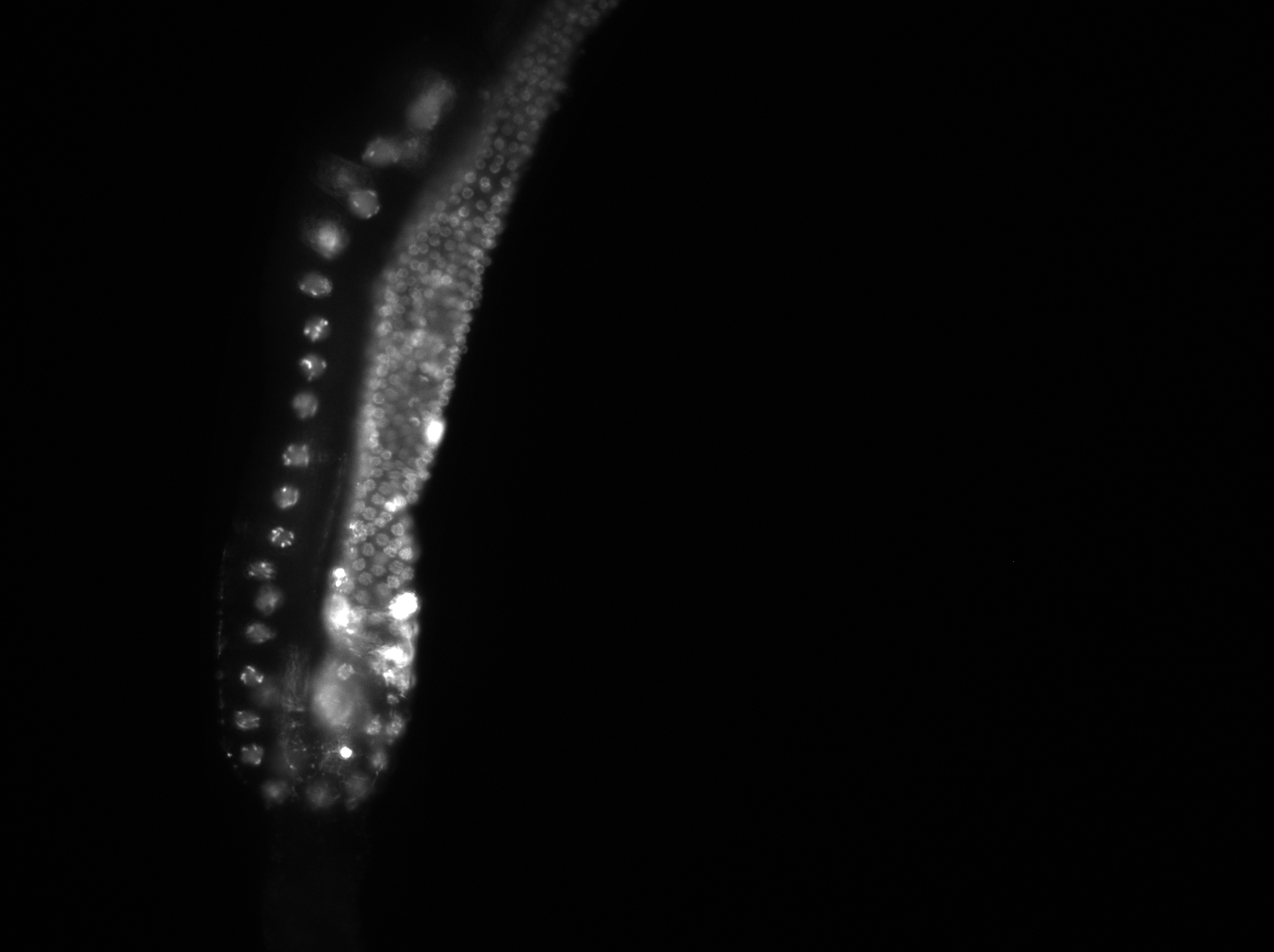

Supplement: Supplementary file 7 — Source Data for Figure 1 [file EMBJ-40-e105280-s006.zip › Figure 1/Figure 1F/F1_pid-1; pid-2; 21U sensor(ON).tif]

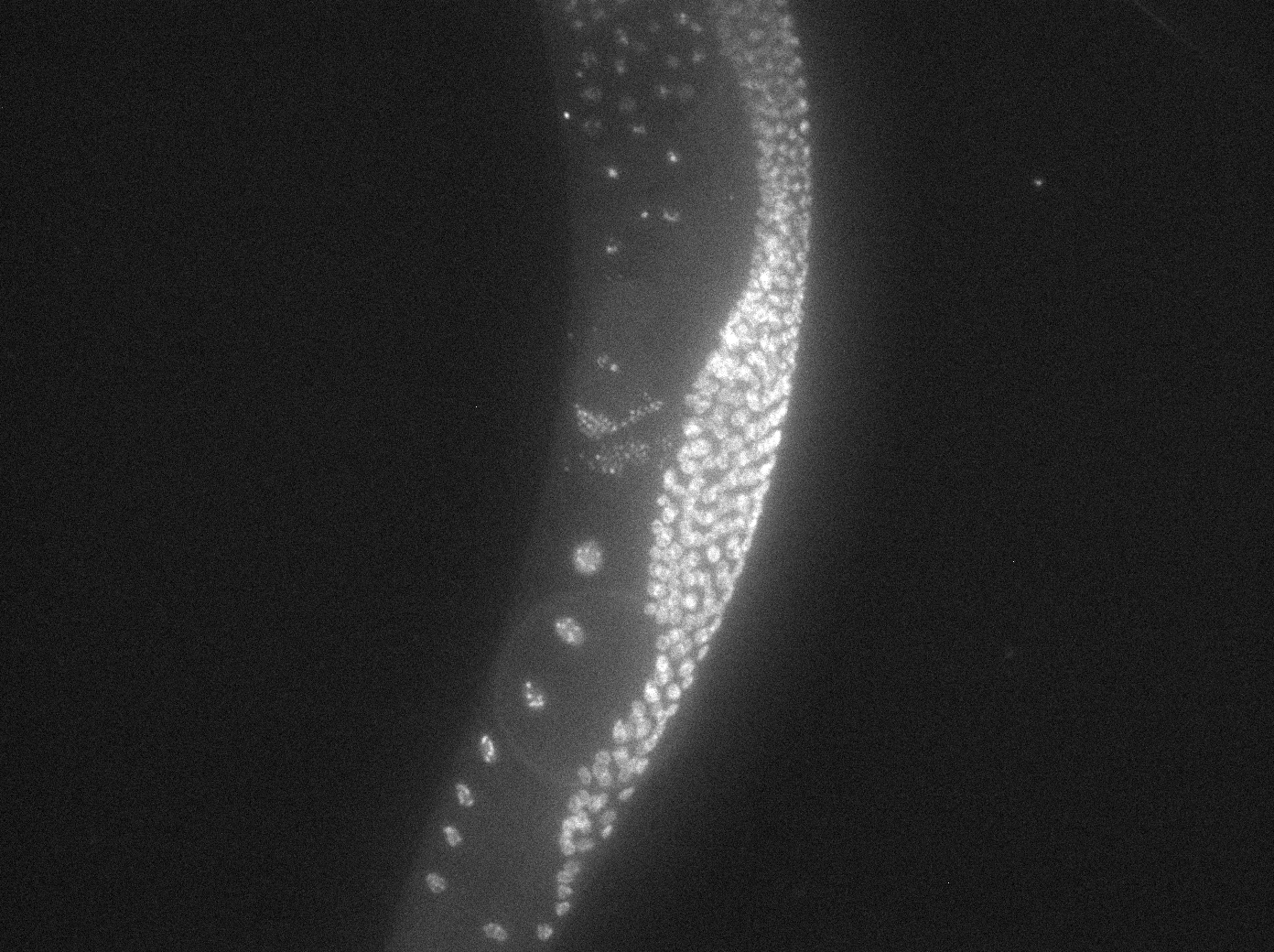

Supplement: Supplementary file 7 — Source Data for Figure 1 [file EMBJ-40-e105280-s006.zip › Figure 1/Figure 1B/pid-2(xf23) 21U sensor(+).tif]

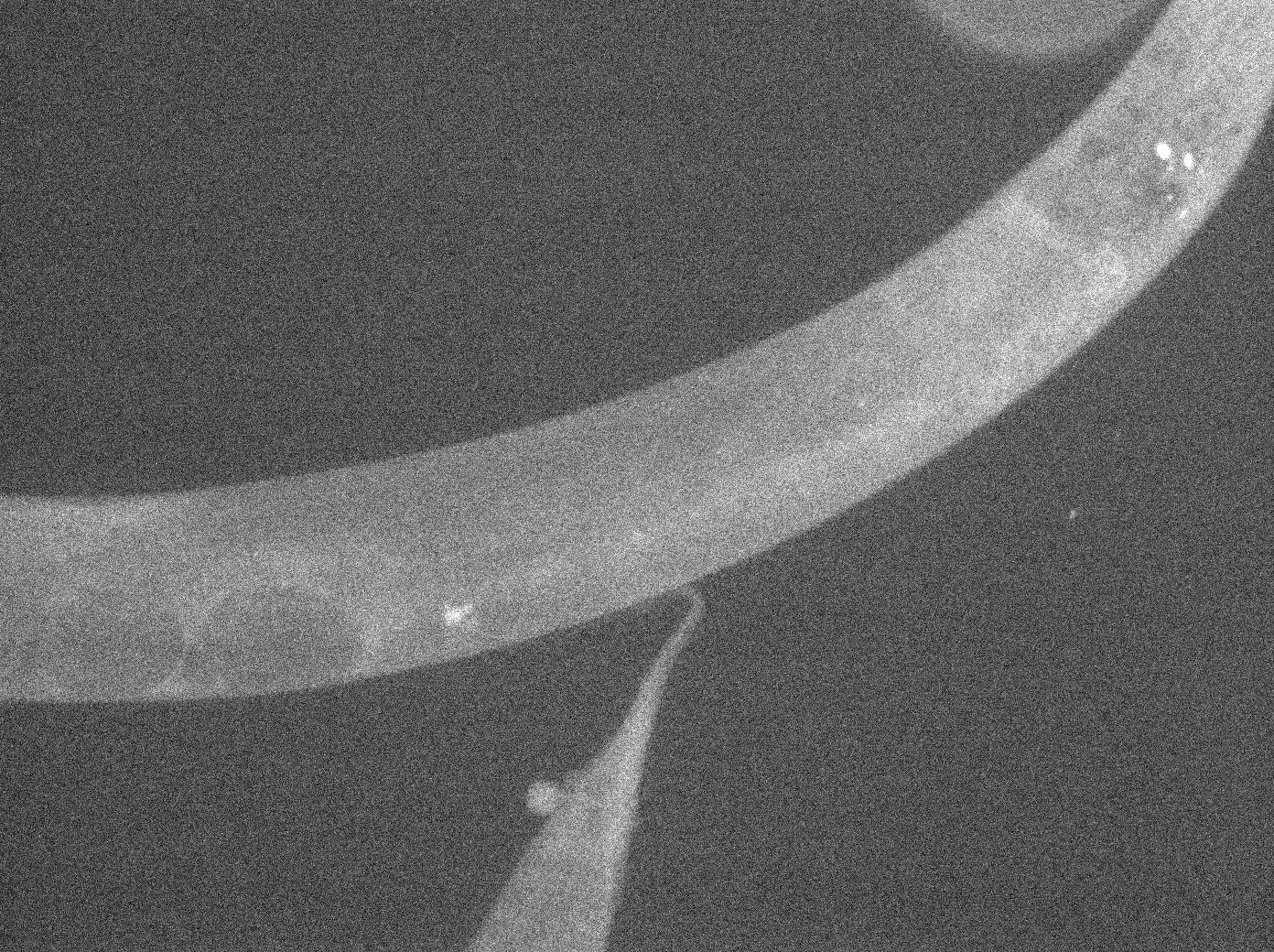

Supplement: Supplementary file 7 — Source Data for Figure 1 [file EMBJ-40-e105280-s006.zip › Figure 1/Figure 1B/pid-2(xf23) 21U sensor(RNAe).tif]

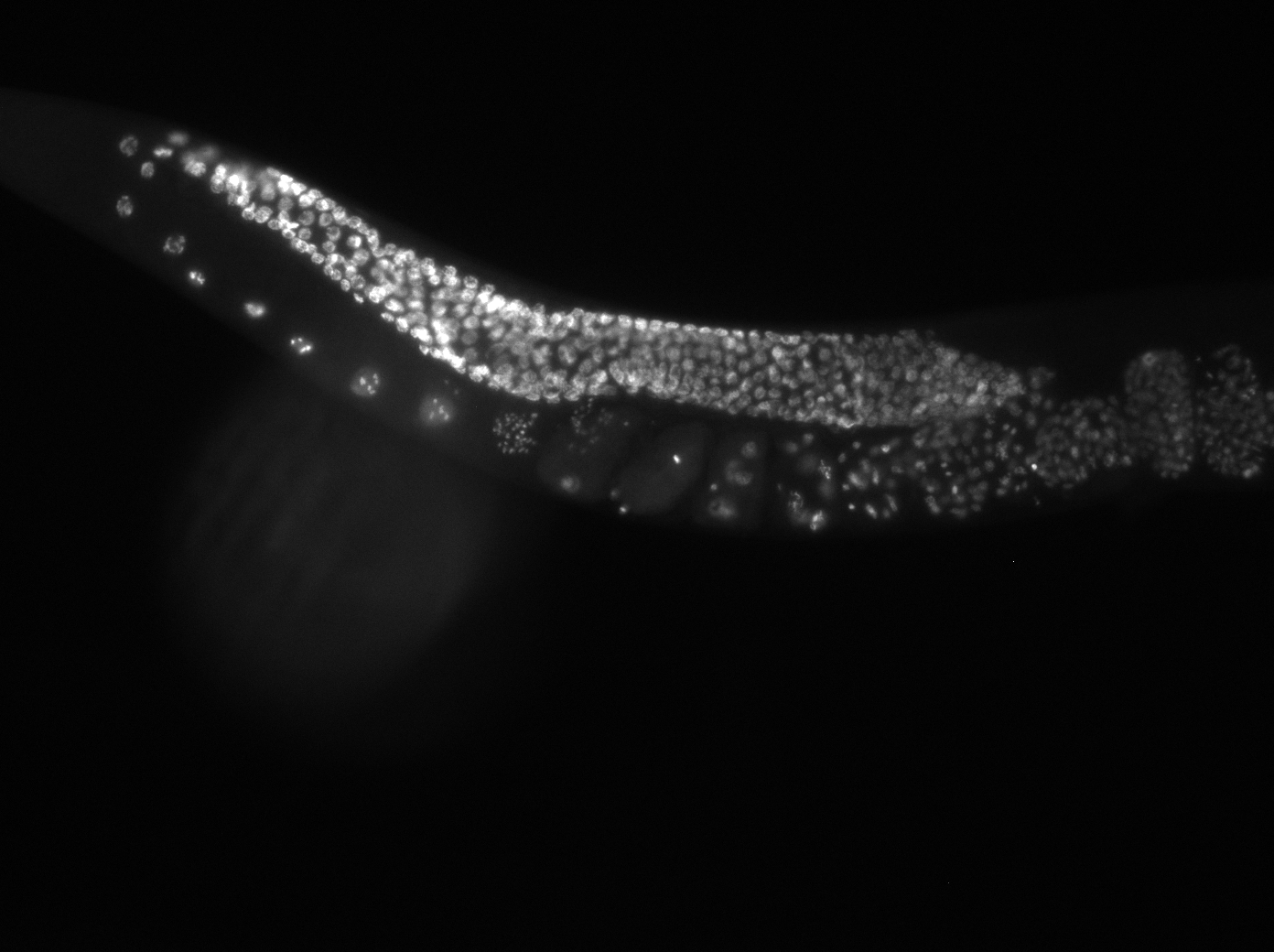

Supplement: Supplementary file 7 — Source Data for Figure 1 [file EMBJ-40-e105280-s006.zip › Figure 1/Figure 1B/mut-7(pk204) 21U sensor(+).tif]

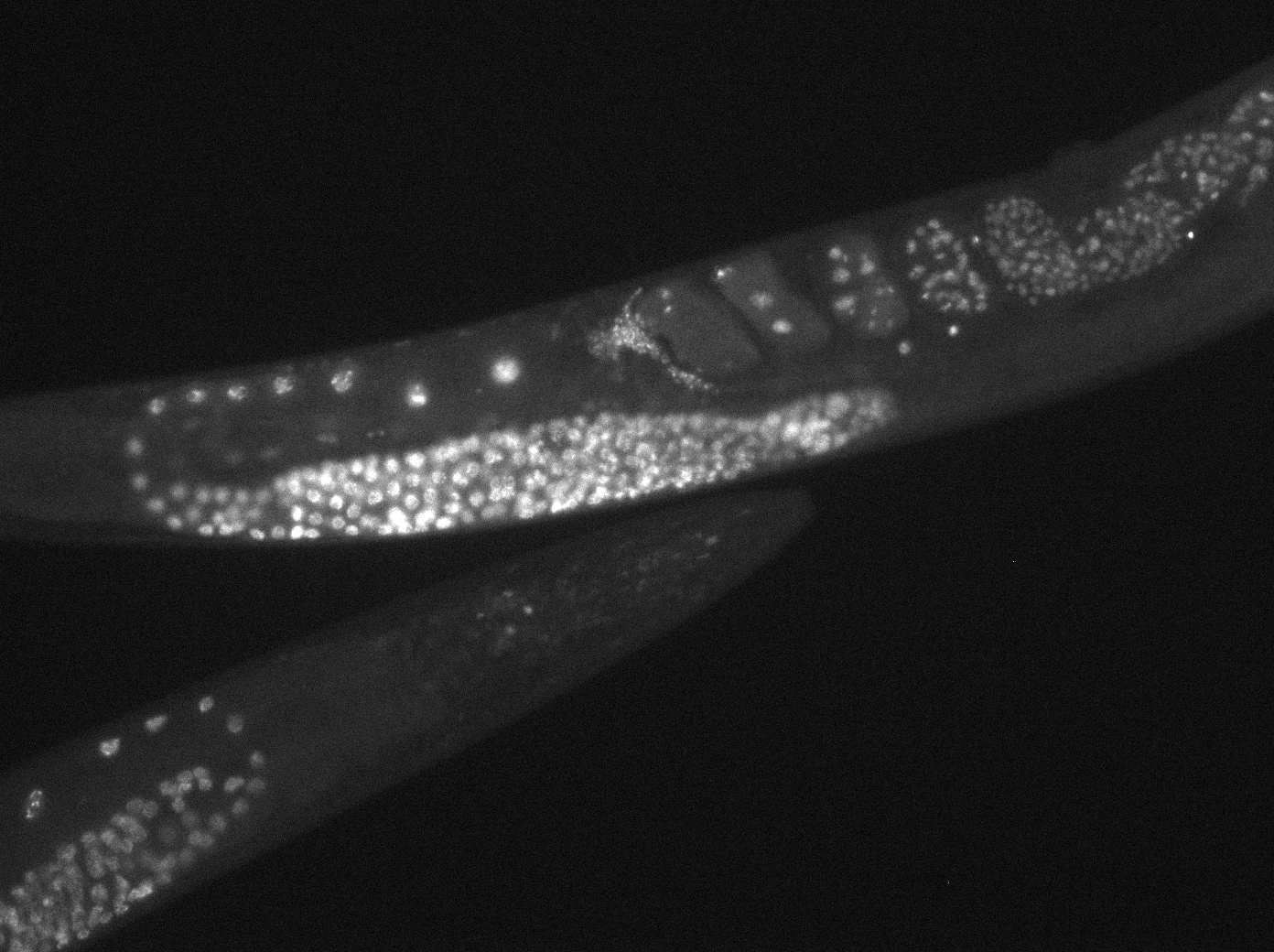

Supplement: Supplementary file 7 — Source Data for Figure 1 [file EMBJ-40-e105280-s006.zip › Figure 1/Figure 1B/pid-2(tm1614) 21U sensor(+).tif]

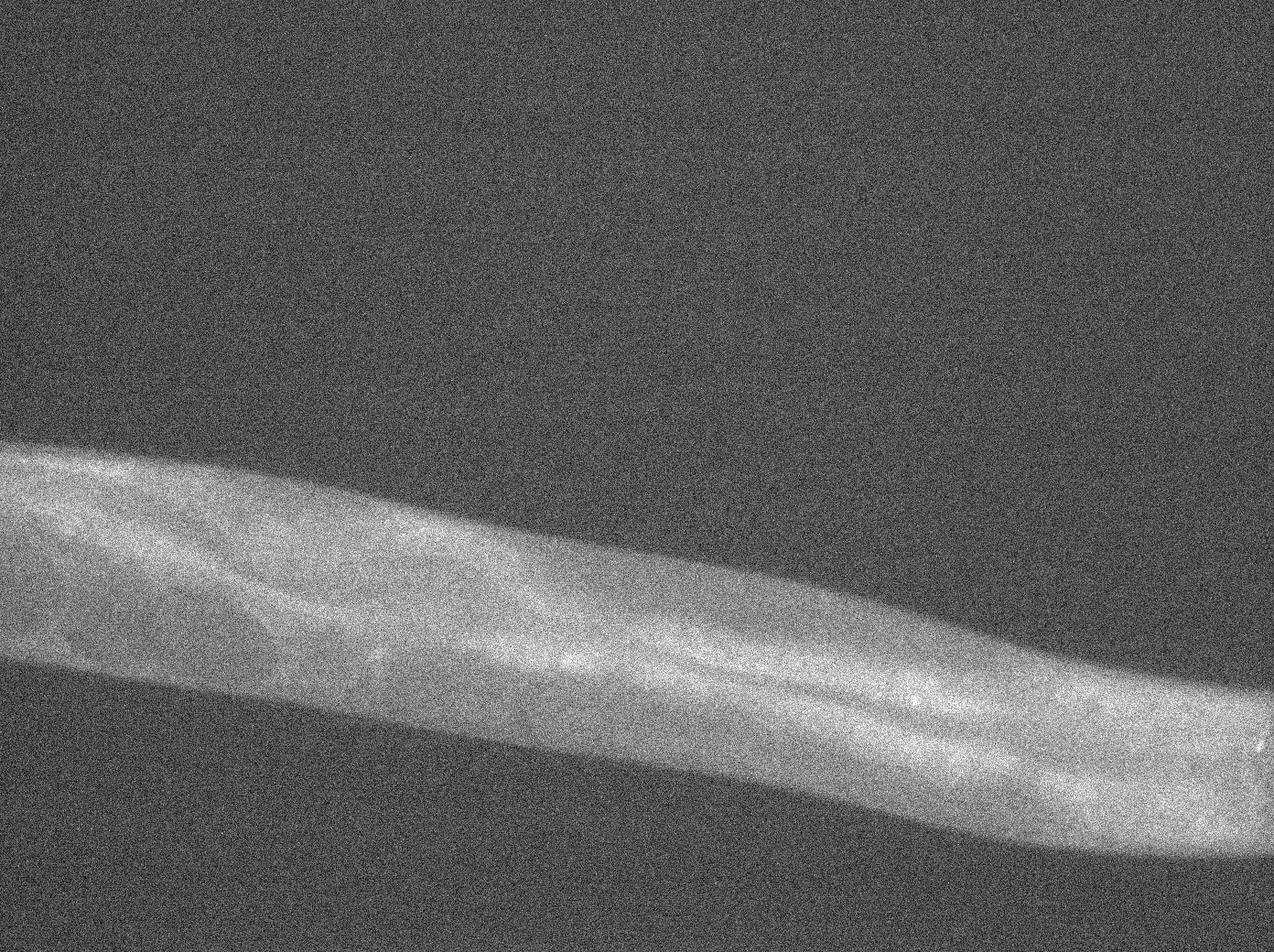

Supplement: Supplementary file 7 — Source Data for Figure 1 [file EMBJ-40-e105280-s006.zip › Figure 1/Figure 1B/pid-2(tm1614) 21U sensor(RNAe).tif]

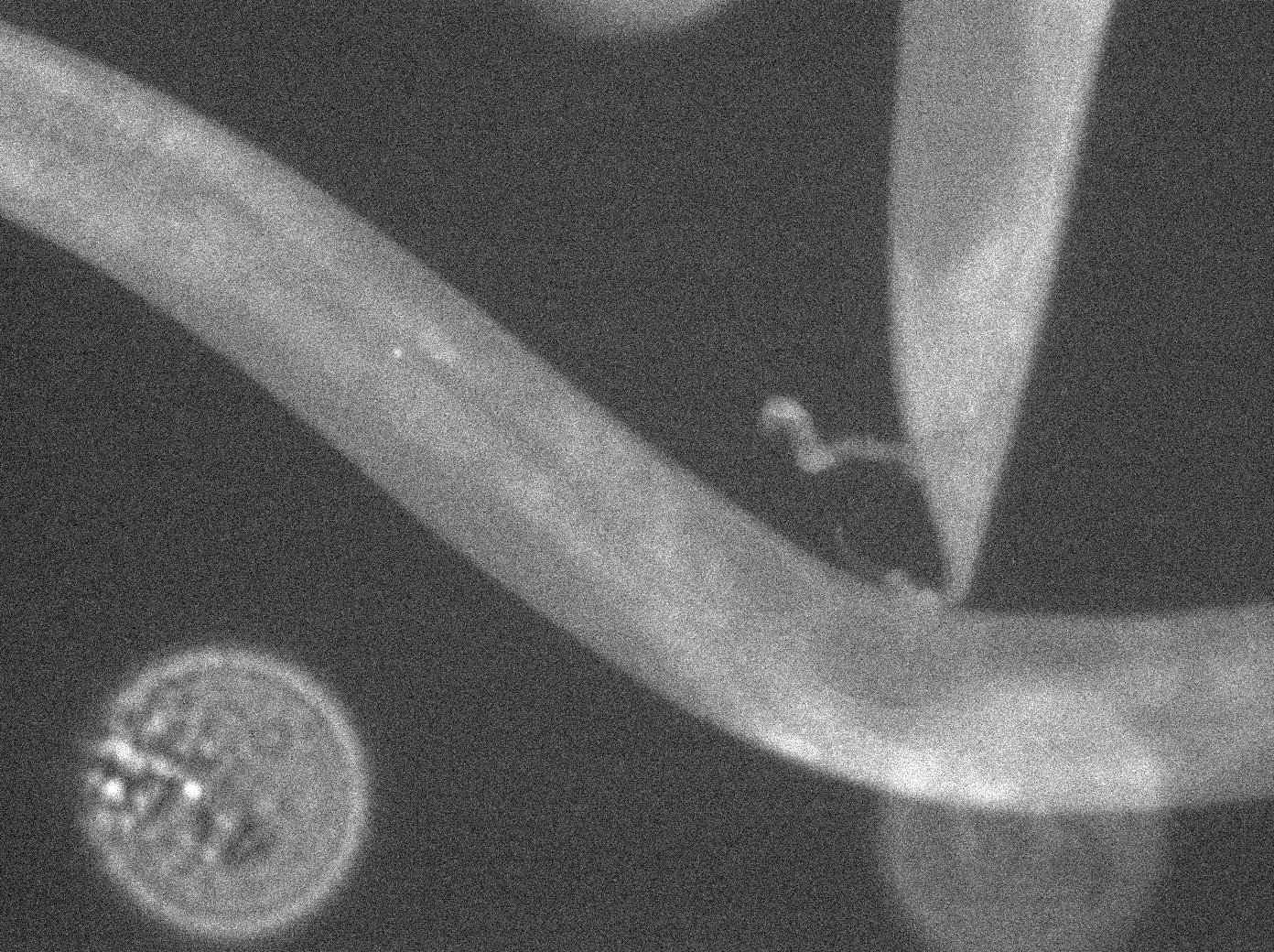

Supplement: Supplementary file 7 — Source Data for Figure 1 [file EMBJ-40-e105280-s006.zip › Figure 1/Figure 1B/prg-1(n4357) 21U sensor(RNAe).tif]

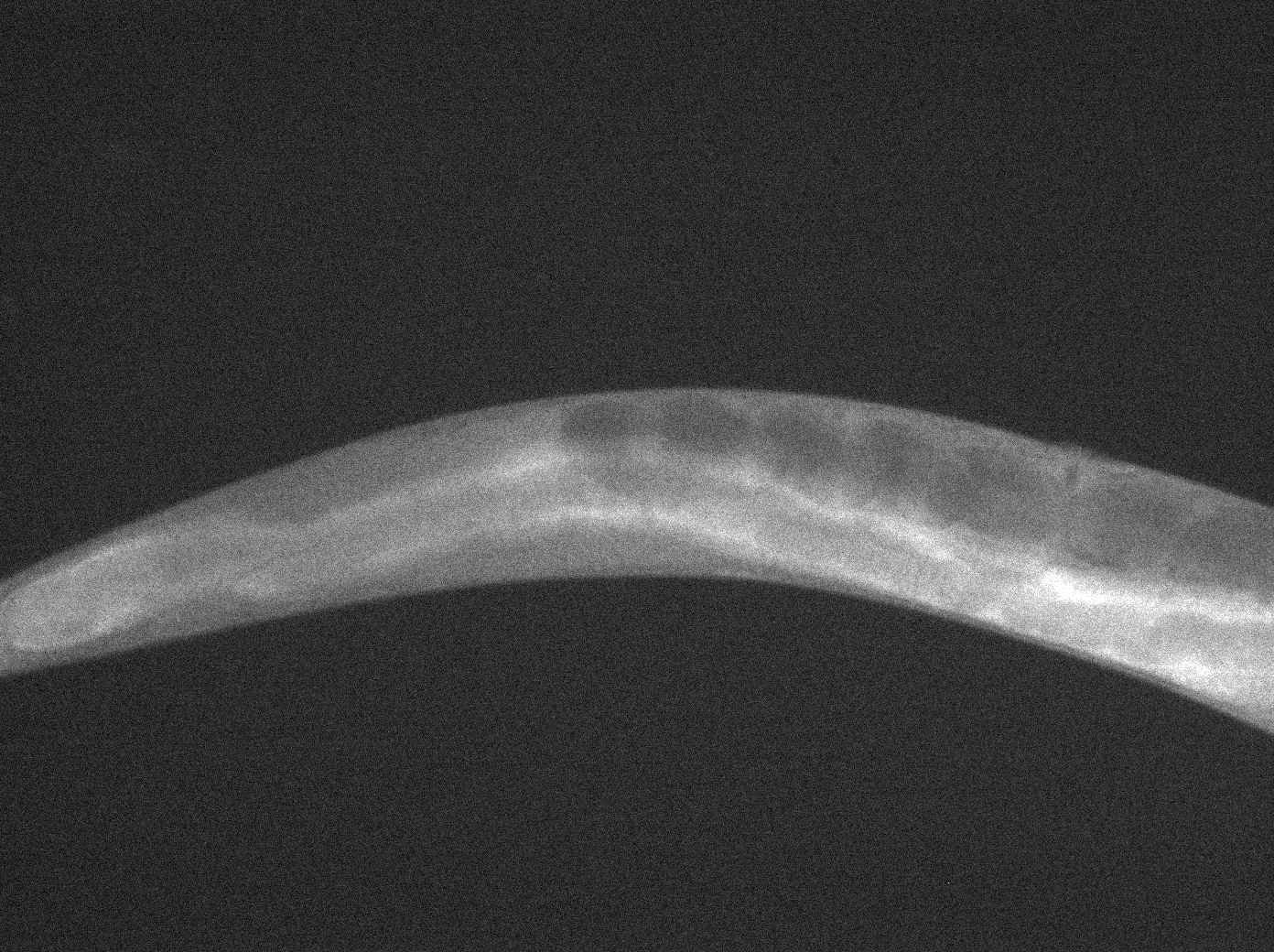

Supplement: Supplementary file 7 — Source Data for Figure 1 [file EMBJ-40-e105280-s006.zip › Figure 1/Figure 1E/F1_pid-1; 21U sensor(OFF).tif]

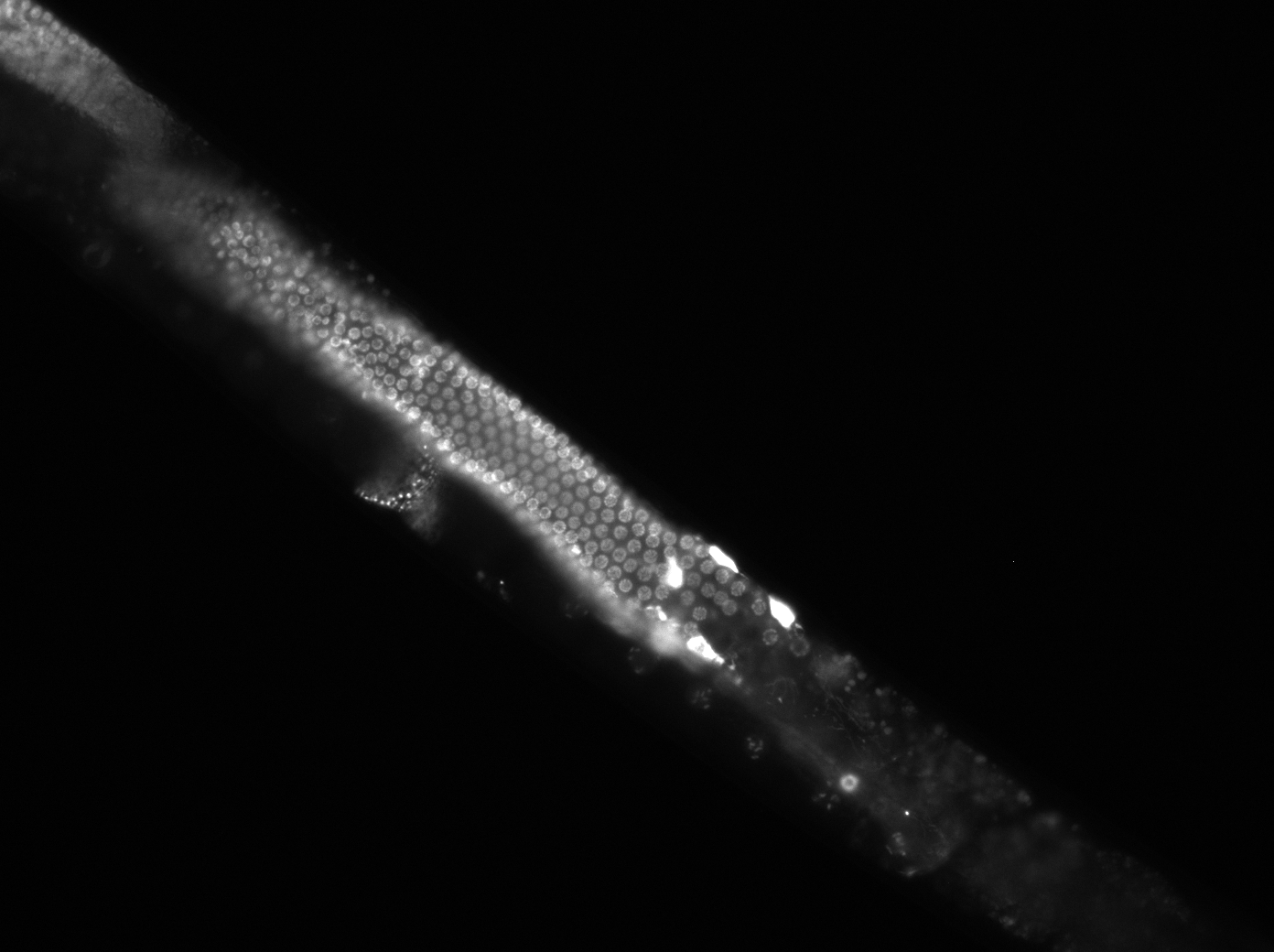

Supplement: Supplementary file 7 — Source Data for Figure 1 [file EMBJ-40-e105280-s006.zip › Figure 1/Figure 1D/F1 mut-7;21U sensor(+) x prg-1.tif]

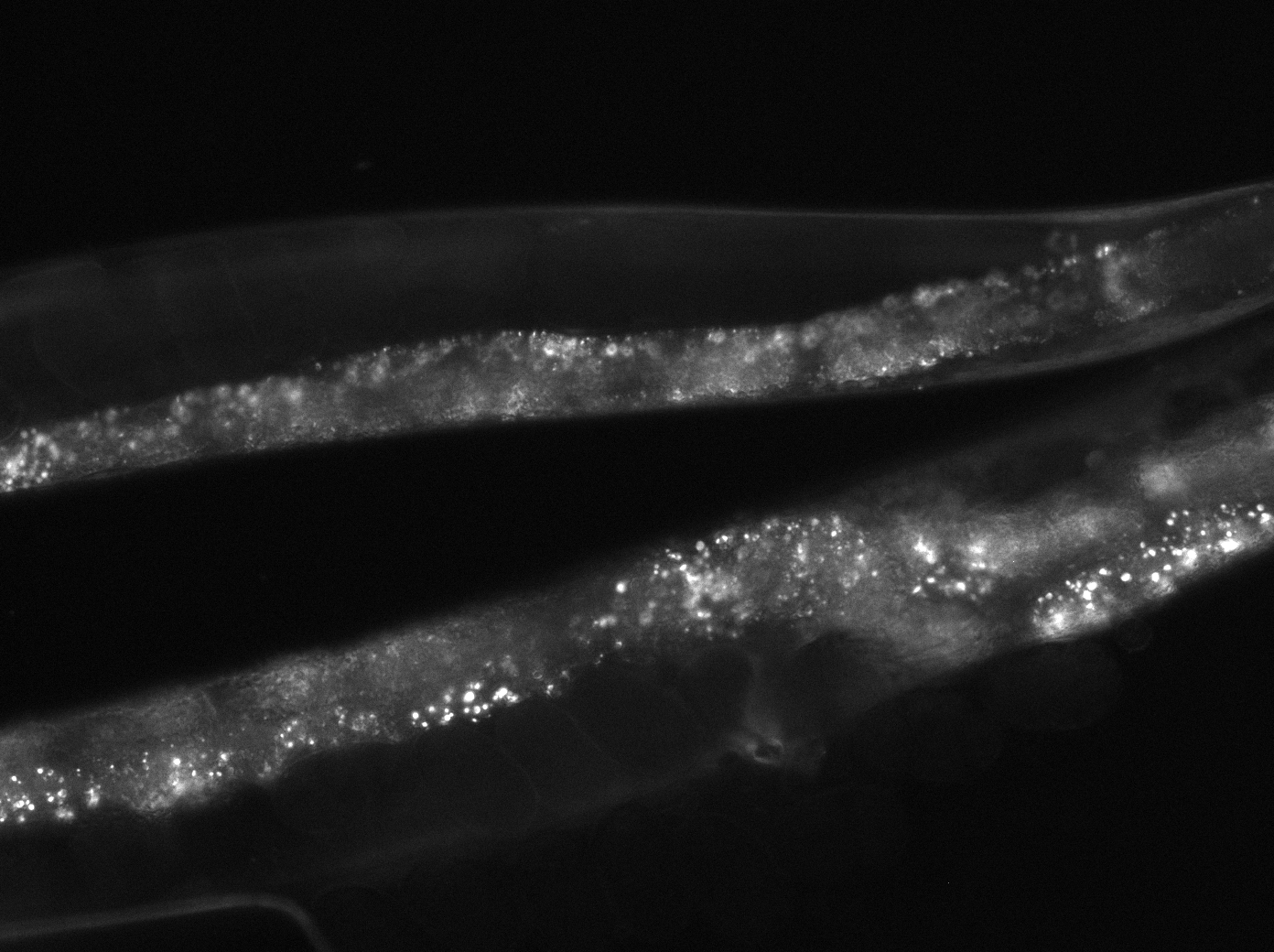

Supplement: Supplementary file 7 — Source Data for Figure 1 [file EMBJ-40-e105280-s006.zip › Figure 1/Figure 1D/F1 mut-7;21U sensor(+) x wildtype.tif]

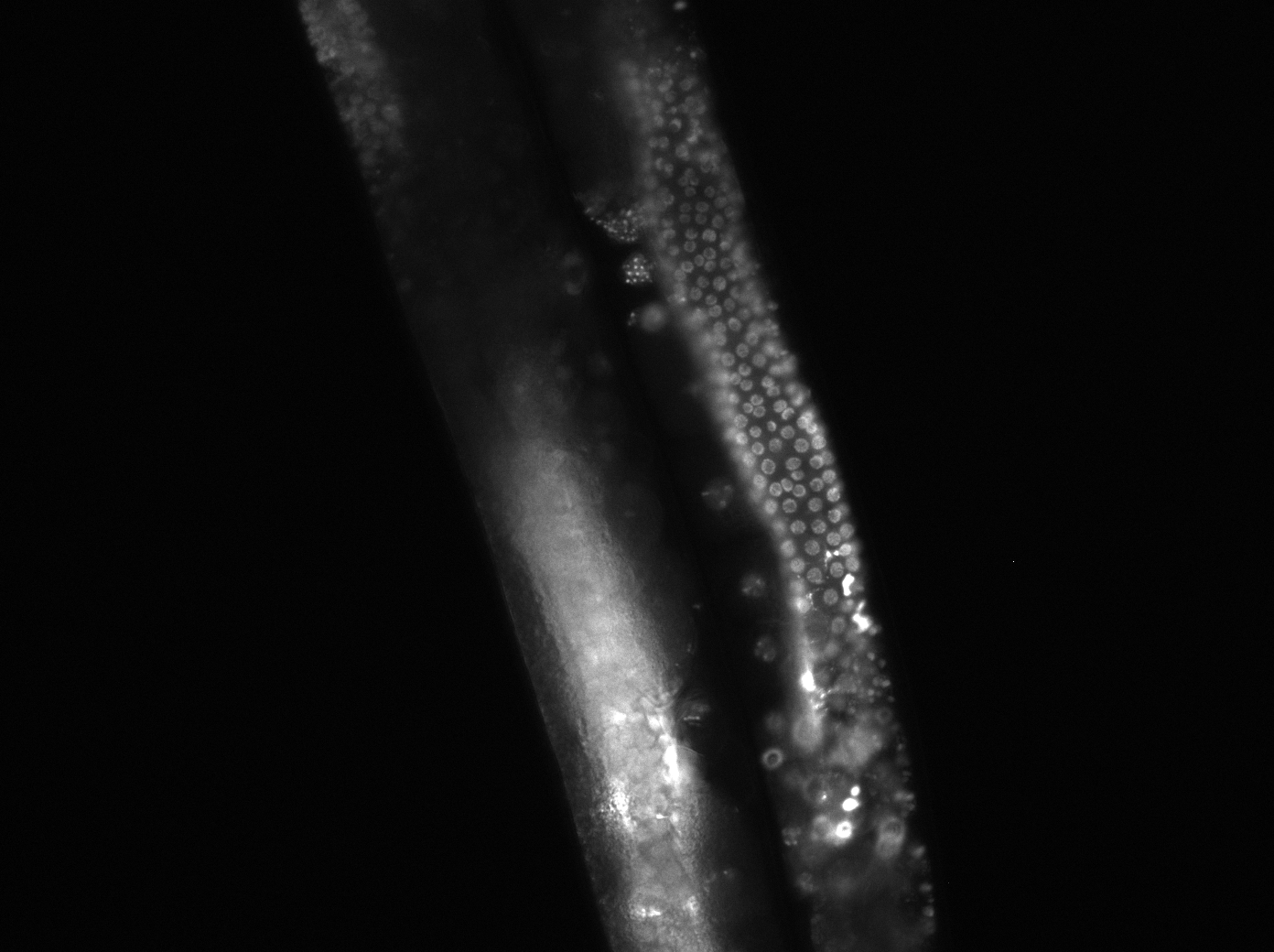

Supplement: Supplementary file 7 — Source Data for Figure 1 [file EMBJ-40-e105280-s006.zip › Figure 1/Figure 1D/F1 mut-7;21U sensor(+) x pid-2(xf23).tif]

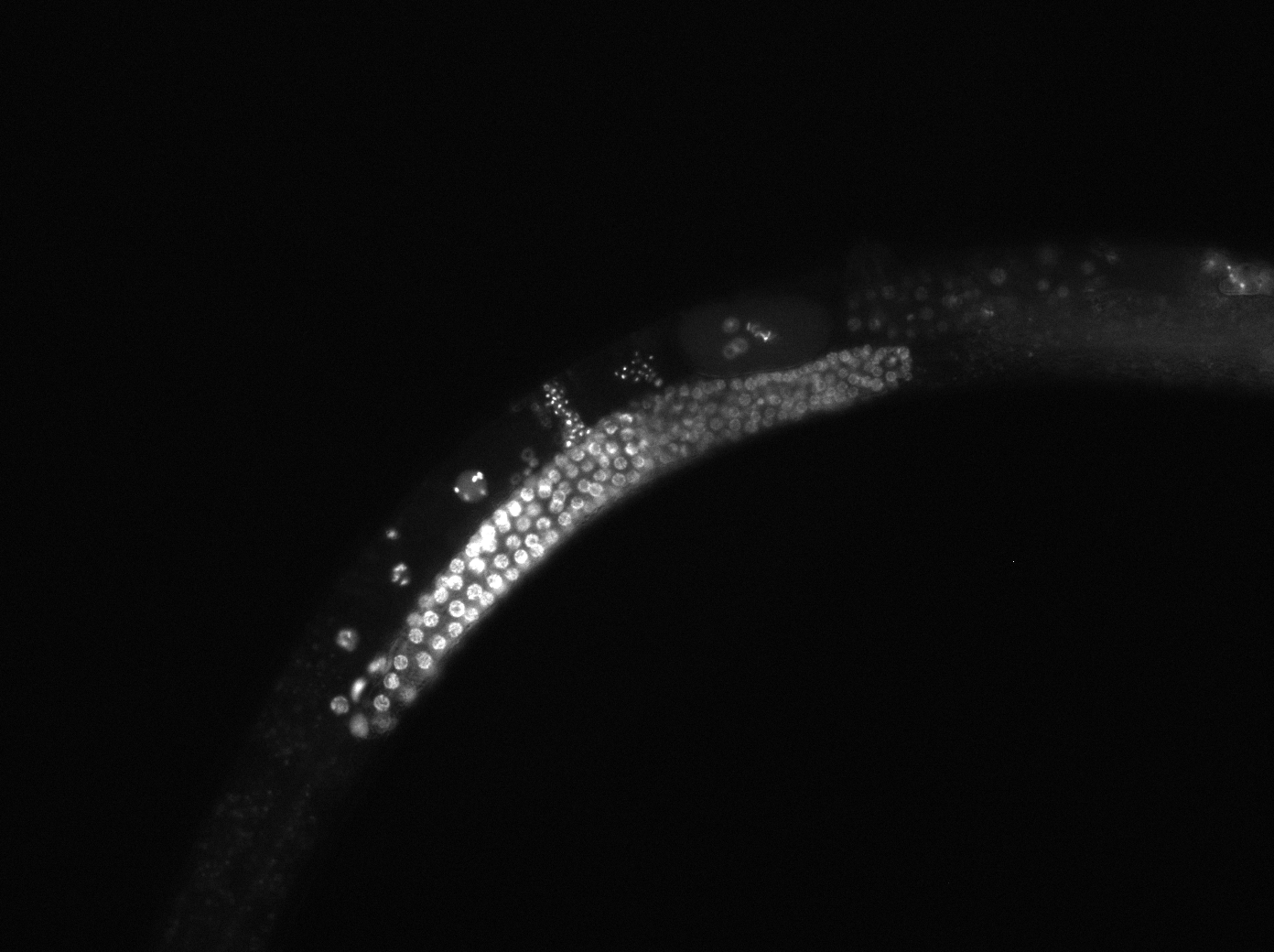

Supplement: Supplementary file 8 — Source Data for Figure 5 [file EMBJ-40-e105280-s007.zip › Figure 5/Figure 5A/mut-7; 21U sensor(+).tif]

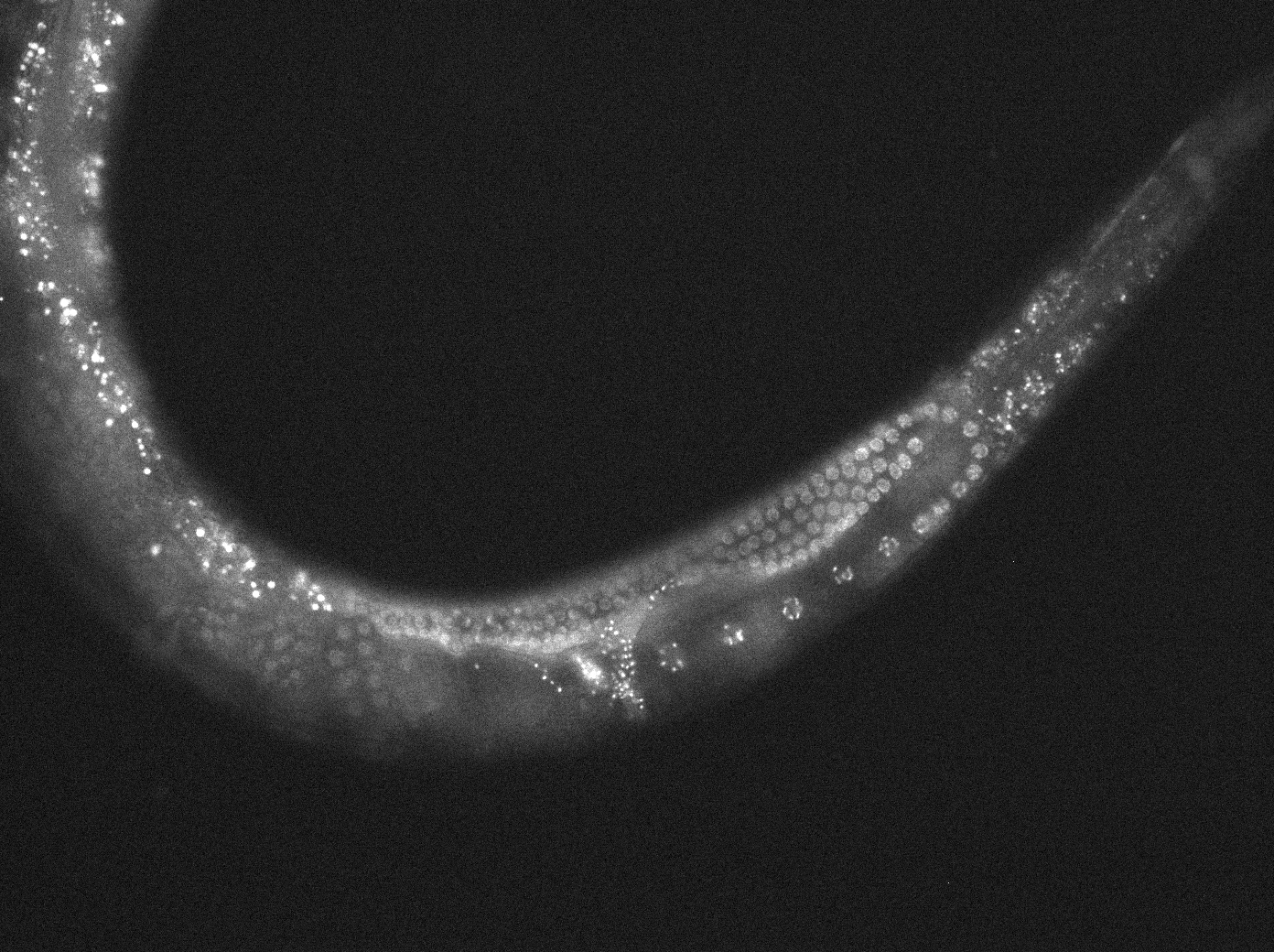

Supplement: Supplementary file 8 — Source Data for Figure 5 [file EMBJ-40-e105280-s007.zip › Figure 5/Figure 5A/pid-4;pid-5; 21U sensor(+).tif]

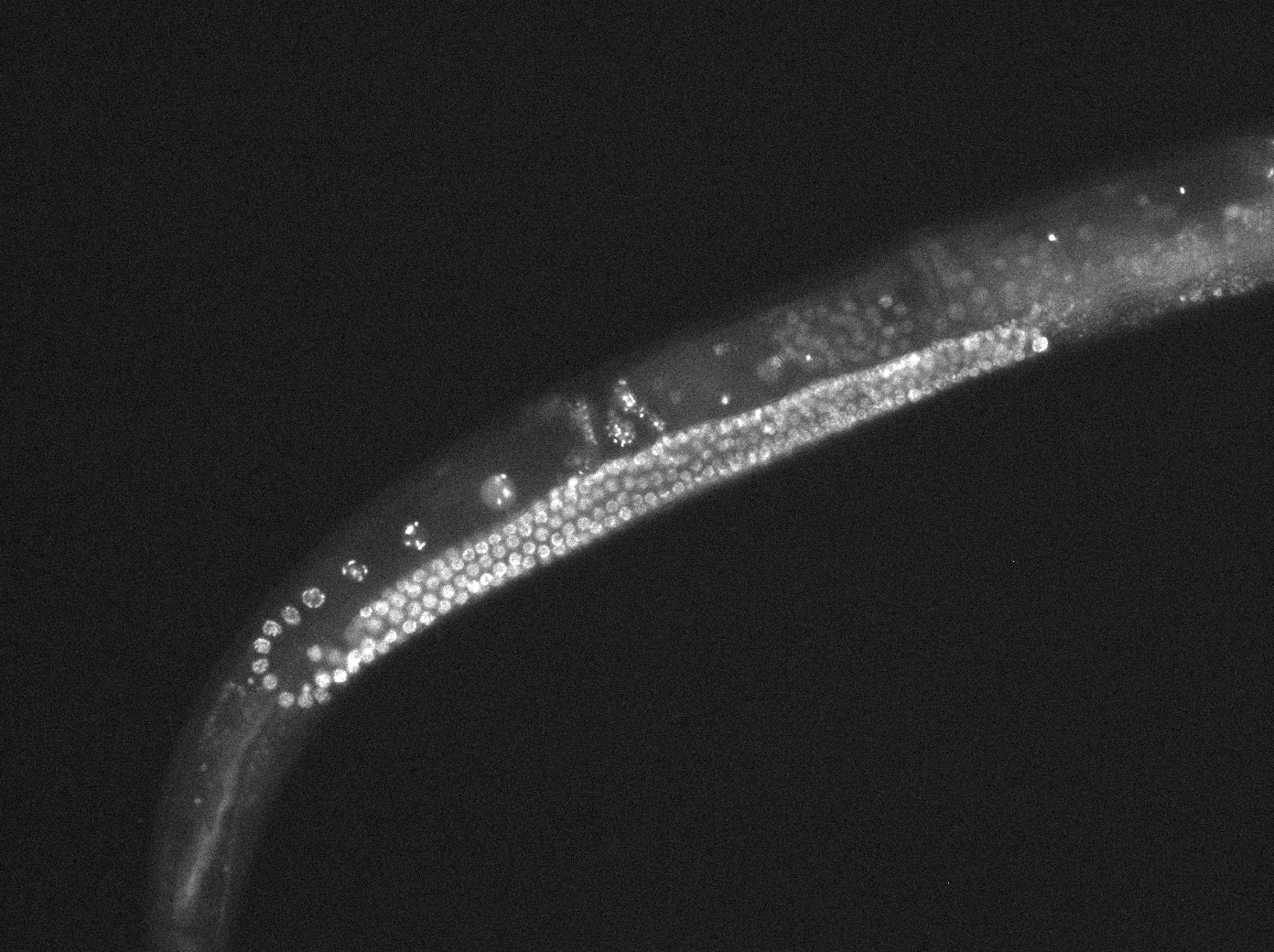

Supplement: Supplementary file 8 — Source Data for Figure 5 [file EMBJ-40-e105280-s007.zip › Figure 5/Figure 5A/pid-2; 21U sensor(+).tif]
