# Supplementary material for: Intrinsically disordered protein PID‐2 modulates Z granules and is required for heritable piRNA‐induced silencing in the Caenorhabditis elegans embryo
Source: EMBO J. 2020 Nov 24;40(3):e105280. doi: 10.15252/embj.2020105280 (PMC7849312; doi:10.15252/embj.2020105280)
Supplement: Supplementary file 9 — Source Data for Figure 6 [file EMBJ-40-e105280-s008.zip › sourceData_Fig6/EMBOJ-2020-105280R1-Annotation_for_source_data-sd-Fig6.rtf]

Some of the files in this folder contain multiple channels. The imaging content of the different channels is:1: GFP2: mTagRFP3: BrightfieldException is Fig 6D:1: mTagRFP2: GFP3: Brighfiled
